# Regulatory Mechanism between Ferritin and Mitochondrial Reactive Oxygen Species in Spinal Ligament-Derived Cells from Ossification of Posterior Longitudinal Ligament Patient

**DOI:** 10.3390/ijms24032872

**Published:** 2023-02-02

**Authors:** Jong Tae Kim, Yonggoo Kim, Ji Yeon Kim, Seungok Lee, Myungshin Kim, Dong Wook Jekarl

**Affiliations:** 1 Department of Neurosurgery, Incheon St. Mary’s Hospital, College of Medicine, The Catholic University of Korea, Seoul 06591, Republic of Korea; 2 Department of Laboratory Medicine, Seoul St. Mary’s Hospital, College of Medicine, The Catholic University of Korea, Seoul 06591, Republic of Korea; 3 Department of Laboratory Medicine, Incheon St. Mary’s Hospital, College of Medicine, The Catholic University of Korea, Seoul 06591, Republic of Korea

**Keywords:** ossification of posterior longitudinal ligament, RNA-seq, mitochondria, reactive oxygen species, ferritin, osteogenic differentiation, alkaline phosphatase

## Abstract

Primary spinal ligament-derived cells (SLDCs) from cervical herniated nucleus pulposus tissue (control, Ctrl) and ossification of the posterior longitudinal ligament (OPLL) tissue of surgical patients were analyzed for pathogenesis elucidation. Here, we found that decreased levels of ferritin and increased levels of alkaline phosphatase (ALP), a bone formation marker, provoked osteogenesis in SLDCs in OPLL. SLDCs from the Ctrl and OPLL groups satisfied the definition of mesenchymal stem/stromal cells. RNA sequencing revealed that oxidative phosphorylation and the citric acid cycle pathway were upregulated in the OPLL group. SLDCs in the OPLL group showed increased mitochondrial mass, increased mitochondrial reactive oxygen species (ROS) production, decreased levels of ROS scavengers including ferritin. ROS and ferritin levels were upregulated and downregulated in a time-dependent manner, and both types of molecules repressed ALP. Osteogenesis was mitigated by apoferritin addition. We propose that enhancing ferritin levels might alleviate osteogenesis in OPLL.

## 1. Introduction

Ossification of the posterior longitudinal ligament (OMIM #602475, OPLL) is characterized by pathologic ossification of a ligament that lies vertically on the posterior side of the spinal body. The progression of ossification compresses the spinal cord and nerve root at the cervical level, leading to myeloradiculopathy and various neurological symptoms [1]. The prevalence of the disease has been reported to range from 0.4 to 3.0% in Asia and from 0.1 to 1.7% in Europe and the United States [2,3,4]. The current treatment involves pain control by medical therapeutics and surgical decompression by cervical laminoplasty [5,6]. Osteogenesis of mesenchymal stem/stromal cells (MSCs) involves various genes [7,8,9,10,11]. A phenotypic study has revealed that mesenchymal stem/stromal cells from OPLL patients are already committed to the osteogenic lineage [12,13,14,15].

Ligament-derived cell including spinal ligament-derived cells (SLDCs) share similar phenotypic and biological features with mesenchymal stem/stromal cells [16,17]. SLDCs show comparable features of stemness and differentiation potentials including osteogenic, chondrogenic, and adipogenic potentials. However, there are differences in the degree of clonogenicity and proliferation potential between cell populations [18,19].

Reactive oxygen species (ROS), such as superoxide anions and hydrogen peroxide, are associated with redox signaling or oxidative stress [20]. The main source of ROS within cells is mitochondria, and mitochondrial ROS production is correlated with elevated mitochondrial membrane potential (ΔΨ_M_) [21,22]. The electron transport chain in the mitochondrial inner membrane, which consists of five complexes and generates the membrane potential. Iron (Fe) produces ROS by the Fenton reaction [23,24,25,26], and carbonyl cyanide m-chlorophenyl hydrazone (CCCP) induces ROS production via mitochondrial depolarization [26,27,28,29,30].

Adverse effects of ROS can be mitigated by intricate mechanisms of ROS-scavenging enzymes, such as superoxide dismutase (SOD), glutathione peroxidase (GPX), and catalase [19,20,21,22]. Autophagy is a catabolic process within cells that is induced under cellular stresses such as ROS accumulation to prevent further cellular damage [29,30,31,32].

Ferritin is a multimeric protein with 24 subunits of heavy and light chains that constitute a cage with a cavity for iron storage [33,34,35,36]. Ferritin inhibits osteogenesis within cells though its ferroxidase activity and inhibits alkaline phosphatase and osteocalin, which are related to osteogenic differentiation [37,38]. In contrast, ferritin promotes resistance to ROS via its anti-ROS scavenging function. Ferritin expression is induced by cellular iron, ROS, and ROS-inducing agents such as hydrogen peroxide and rotenone [37,38].

High-throughput mRNA sequencing (RNA-seq) provides the ability to measure the whole transcriptome via massive read production [39,40]. Differentially expressed genes (DEGs), new genes, alternative splice isoforms, and rare transcripts can be analyzed by RNA-Seq [41,42,43,44,45,46].

In this study, we found that primary SLDCs from OPLL patients showed greater osteogenic potential than SLDCs from cervical herniated nucleus pulposus patients, which constituted the control group (Ctrl). Increased intracellular ROS (icROS) from mitochondria resulted in repression of alkaline phosphatase (ALP). Within SLDCs, ROS and ferritin were related in a time-dependent manner, and both molecules repressed ALP. A low ferritin level abrogated the repression of ALP in the OPLL group compared to the Ctrl group.

## 2. Results

### 2.1. SLDCs Exhibited Properties of Mesenchymal Stem/Stromal Cells

**Figure 1:**
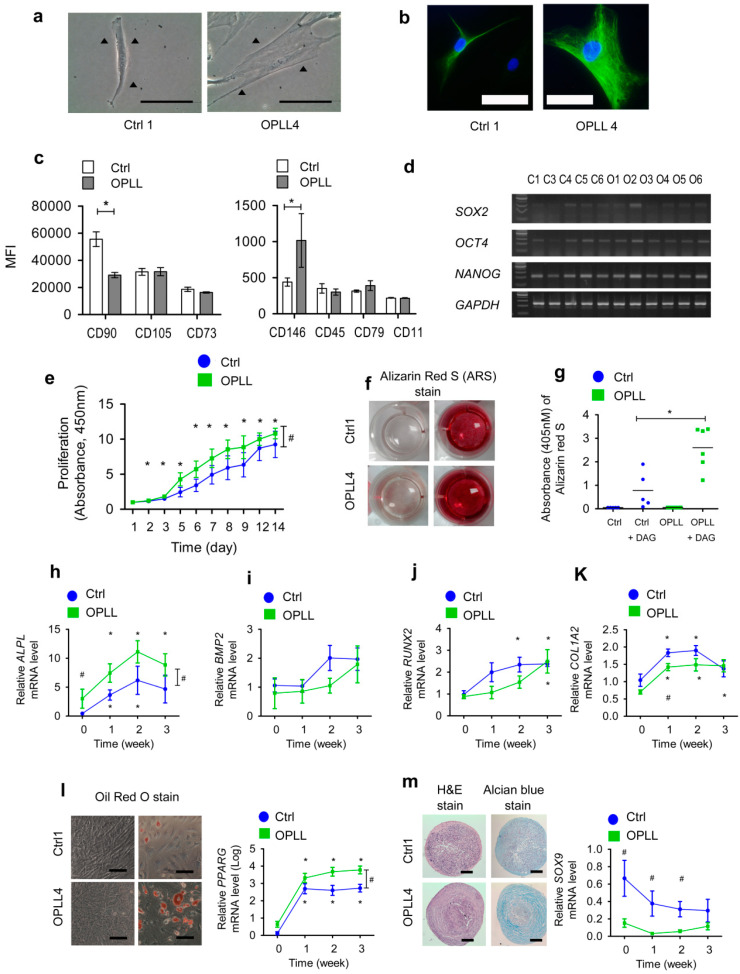
Phenotypic features of primary spinal ligament-derived cells (SLDCs) from the control (Ctrl) and OPLL groups. Unless otherwise noted, the representative data were obtained from triplicate experiments. (**a**, left) Live cell images of primary SLDCs from Ctrl1 and (**a**, right) from OPLL4 indicated by black triangles using an inverted microscope (×400). (**b**) Live cell images of anti-nestin antibody (green) staining and DAPI nuclear counterstaining (blue) for Ctrl1 (**b**, left) and OPLL4 (**b**, right) captured by fluorescence microscopy. (**c**) An immunophenotypic study to define mesenchymal stem cells was performed. CD90, CD105, CD73, CD45, CD79, CD11, and CD146 expression in the Ctrl (n = 5) and OPLL (n = 6) groups was analyzed. (**d**) Gene expression was studied for *SOX2, OCT4,* and *NANOG*, which are related to the stemness of mesenchymal stem cells. (**e**) The proliferation of cells was measured by incubating SLDCs of the Ctrl and OPLL groups with 10 µL of CCK-8 reagent for 4 h from Day 0 to Day 14. (**f**) Osteogenic differentiation was performed by induction for three weeks with osteogenic medium (DAG) that included αMEM, 10% fetal bovine serum (FBS), dexamethasone (10 nM), ascorbic acid (250 µM), and β-glycerophosphate (10 mM). The absorbance of dissolved Alizarin red S (ARS) was measured at 405 nM for Ctrl1 and OPLL4. (**g**) ARS staining results after 3 weeks of osteogenic differentiation for the Ctrl (n = 5) and OPLL (n = 6) groups. (**h**–**k**) Gene expression profiles related to osteogenic differentiation were determined by qRT-PCR for (**h**) *ALPL*, (**i**) *BMP2*, (**j**) *RUNX2*, and (**k**) *COL1A2* during the 3 weeks of osteogenic differentiation. (**l**) Cells from Ctrl1 and OPLL4 were subjected to adipogenic differentiation for 3 weeks and then stained with Oil red O followed by *PPARG* gene expression analysis. (**m**) Cells from Ctrl1 and OPLL4 were subjected to chondrogenic differentiation for 3 weeks and then stained with Alcian blue followed by *SOX9* gene expression analysis. Throughout, the data are presented as the mean ± SEM. * *p* < 0.05 within groups; ^#^ *p* < 0.05 between groups. Scale bars: white, 50 μm; black, 100 μm.

Primary SLDCs from five Ctrl and six OPLL surgical patients that adhered to the bottom of the culture plate were isolated (Table 1). The unstained live cell images or nestin-stained cell images revealed that Ctrl1 SLDCs were sharp and spindle-shaped with a small to medium size, whereas OPLL4 SLDCs were round and cuboidal with a medium to large size (Figure 1a,b). The immunophenotypic study using flow cytometry showed that both groups were positive for CD90, CD105, and CD73 but negative for CD45, CD79, and CD11 (Figure 1c). Reverse transcription polymerase chain reaction analysis of genes related to pluripotency showed that the genes *SOX2*, *OCT4*, and *NANOG* were also expressed in both groups (Figure 1d) [47]. SLDCs from the OPLL group showed significantly greater proliferation capacity than SLDCs from the Ctrl group from Day 5 to Day 12, and this effect gradually plateaued (Figure 1e).

### 2.2. SLDCs from the OPLL Group Showed Enhanced Osteogenic Differentiation

Osteogenic differentiation was tested using a combination of dexamethasone (D), ascorbic acid (A), and β-glycerophosphate (G). After three weeks, the absorbance of dissolved Alizarin Red S (ARS) was measured for ossification quantification [3]. The DAG combination resulted in greater osteogenic differentiation than the DG, DA, AG, D, A, and G treatments after 2 weeks of culture (Supplementary Figure S2). Osteogenic differentiation was increased in SLDCs from the OPLL group (Figure 1f,g and Supplementary Figure S2a,b). Relative gene expression analysis by quantitative real-time polymerase chain reaction (qRT-PCR) with SYBR Green showed that *ALPL* gene expression was initially higher in the OPLL group than in the Ctrl group, whereas *BMP2*, *RUNX2*, and *COL1A2* mRNA levels showed little differences (Figure 1h–k). *ALPL* gene expression was increased in both groups at 1, 2, and 3 weeks during osteogenic differentiation (Figure 1h).

Adipogenic differentiation analysis revealed that the OPLL group showed more lipid droplets than the Ctrl group according to Oil Red O staining (Figure 1l, Supplementary Figure S2c). *PPARG* gene expression was increased in the OPLL group throughout differentiation. Chondrogenic differentiation was assessed by pellet formation for 3 weeks (Figure 1m and Figure S1b). The SLDCs from both groups demonstrated chondrogenic differentiation with lower levels of *SOX9* in the OPLL group than in the Ctrl group.

Altogether, the results indicated that the SLDCs from the Ctrl and OPLL groups exhibited properties of mesenchymal stem/stromal cells. The SLDCs from the OPLL group showed increased proliferation capacity and enhanced ossification and adipogenic differentiation. To understand the mechanism, transcriptome analysis by RNA sequencing (RNA-seq) was performed.

### 2.3. Enhanced TCA Cycle and Oxidative Phosphorylation Pathway in the OPLL Group

The RNA-seq results for osteogenesis, adipogenesis, and chondrogenesis genes coincided with the qRT-PCR results shown in Figure 1 (Supplementary Figure S3a–c). A total of 85 million reads to 101 million reads (Supplementary Table S1) were produced for the Ctrl and OPLL groups (Supplementary Table S2) [42]. Most transcripts had FPKM values below 1 (Supplementary Table S3 and Supplementary Figure S4a–c) [40]. The most abundant transcripts are listed with the gene names in Supplementary Tables S4 and S5.

**Figure 2:**
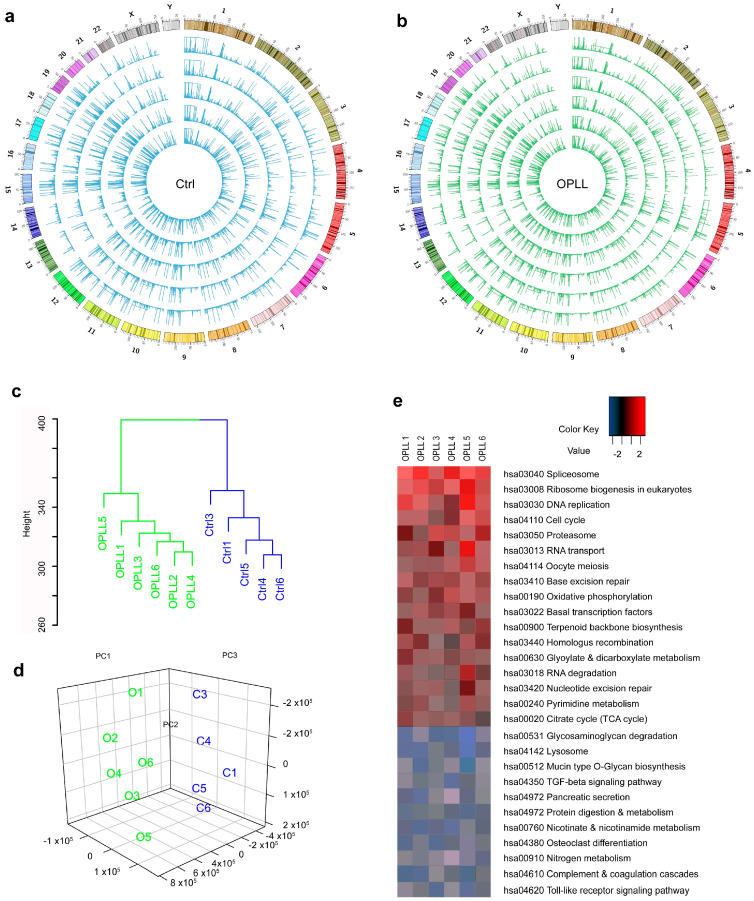
RNA-seq analysis results for spinal ligament-derived cells (SLDCs) from control (Ctrl, n = 5) and OPLL (n = 6) patients. (**a**,**b**) Circos plot of the global gene expression patterns for the Ctrl and OPLL groups, which showed similar patterns. The ideograms for chromosomes 1 to X are plotted in the outer circle, and the expression patterns are plotted under the ideograms except for the Y chromosome for which the pattern is inside the ideogram. (**c**) Hierarchical clustering analysis of the raw read counts using Euclidean distance. The data were obtained from the Ctrl (blue) and OPLL (green) groups. (**d**) Principal component analysis was performed to cluster patients based on major components. The 3D plot of the Ctrl (blue) and OPLL (green) groups was created with principal components (PCs) 1, 2, and 3. (**e**) Pathway analysis was performed using GAGE gene set enrichment analysis. Differentially expressed genes identified by the transcriptome analysis were mapped to KEGG pathways. Upregulated (red) and downregulated (blue) pathways in the OPLL group were plotted. FPKM, fragments per kilobase of exon per million fragments mapped; PC, principal component.

The global expression (FPKM) values are plotted in a Circos plot (Figure 2a,b) and seemed to be similar between the Ctrl and OPLL groups. Each of the samples demonstrated that the correlation coefficient ranged from 0.78 to 0.82 (Supplementary Table S6, Supplementary Figure S5a). However, hierarchical clustering analysis by means of Euclidean distance (Figure 2c) and principal component analysis by means of principal components 1, 2, and 3 clustered the Ctrl and OPLL groups (Figure 2d).

Intersection of the differentially expressed genes (DEGs) with EdgeR, Limma, and DESeq [43,44] resulted in 392 DEGs (Supplementary Figure S5b and Supplementary Tables S7–S9). For these 392 genes, gene set enrichment analysis (GSEA) and an analysis with the Database for Annotation, Visualization and Integrated Discovery (DAVID) were performed to correlate the genes with their functions defined by the Genome Ontology Consortium (Supplementary Tables S10 and S11) [45,46]. Whole gene expression was analyzed by pathway analysis using GAGE gene set enrichment (Figure 2e). In the OPLL group, genes related to the TCA cycle and the oxidative phosphorylation pathway were overexpressed, while genes related to lysosomes were downregulated (Supplementary Tables S12 and S13) [47,48,49,50].

Altogether, the global gene expression analysis showed a common expression pattern between the Ctrl and OPLL groups. However, differences were found in the clustering, principal component, and pathway analyses. Genes related to the TCA cycle and the oxidative phosphorylation pathway were upregulated. These data led to the hypothesis that mitochondrial activity might be related to OPLL.

### 2.4. Mitochondrial Characteristics and ROS Status

**Figure 3:**
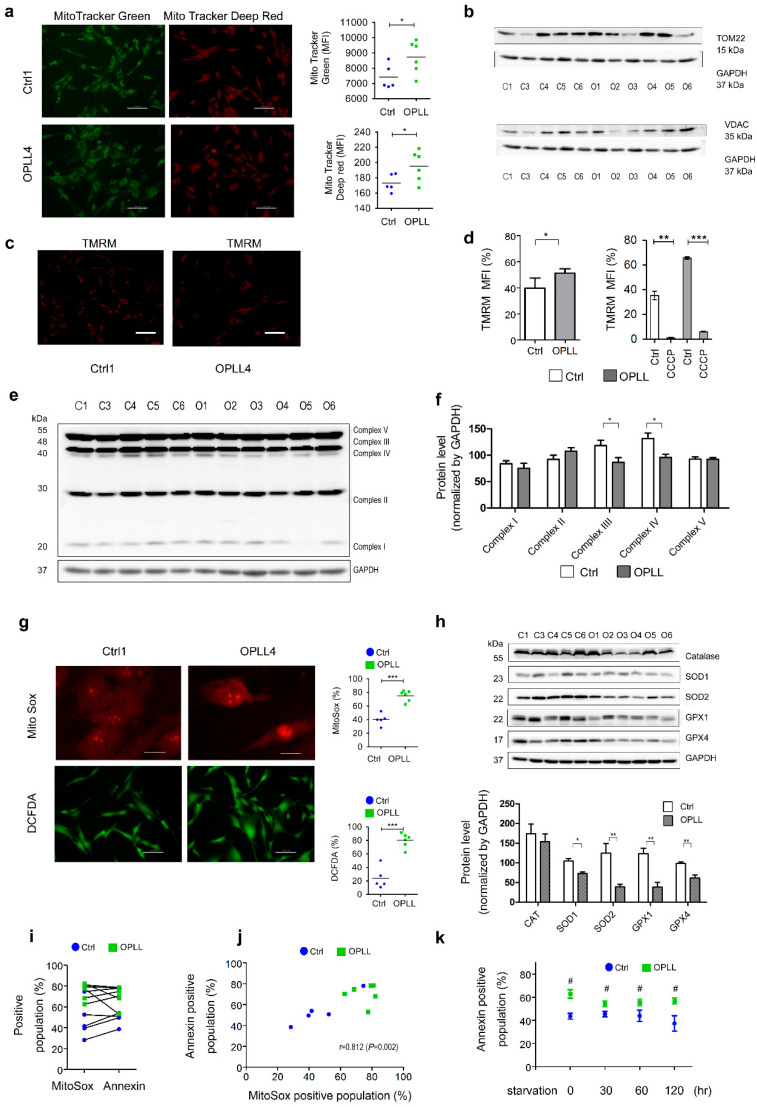
Assessment of mitochondrial mass, membrane potential (ΔΨM), and electron transport chain (ETC) protein complexes. (**a**, left) Live cell images of mitochondria from Ctrl1 and OPLL4 stained using MitoTracker Green (MTG) and MitoTracker Deep Red (MTDR) for mitochondrial mass assessment. (**a**, right) Mitochondrial mass was estimated by fluorescence using 200 nM MTG and 500 nM MTDR for 30 min at 37 °C. One dot implies the mean fluorescence of triplicate results. (**b**) Mitochondrial mass was estimated according to the mitochondrial outer membrane proteins TOM22 and VDAC. (**c**) For ΔΨ_M_ evaluation, live cell images were obtained of Ctrl1 and OPLL4 cells stained with 100 nM tetramethylrhodamine methylester (TMRM) for 30 min. (**d**) The fluorescence levels for the Ctrl and OPLL groups were determined by flow cytometry. The TMRM fluorescence was decreased by CCCP. (**e**) The levels of the mitochondrial ETC complex I~V proteins were assessed by Western blotting. (**f**) Densitogram of the mitochondrial ETC complex normalized to GAPDH. (**g**, left) Representative live cell images of Ctrl1 and OPLL4 were obtained after culturing the cells on cover slips for two days. Superoxide (O_2_^•−^) levels were estimated with 5 μM MitoSox, and intracellular ROS (icROS) levels were estimated with 25 μM DCFDA. (**g**, right) Superoxide (O_2_^•−^) and icROS were measured for all Ctrl and OPLL groups. (**h**, upper) Proteins related to the ROS scavenging system were evaluated. Catalase, SOD 1, SOD 2, GPX 1, and GPX 4 levels of the Ctrl and OPLL groups are shown. (**h**, lower) Densitogram with GAPDH normalization showing that SOD1, SOD2, GPX1, and GPX4 levels were significantly decreased in the OPLL group. (**i**) Superoxide (O_2_^•−^) indicated by MitoSox staining and apoptosis indicated by Annexin V staining were studied by flow cytometry. These cells were measured once. (**j**) The correlation of superoxide (O_2_^•−^) and Annexin V fluorescence was measured simultaneously by flow cytometry. These cells were measured once. (**k**) Starvation of the Ctrl and OPLL groups for apoptosis evaluation using Annexin V by flow cytometry. These cells were measured in triplicate. * *p* < 0.05, ** 0.001 ≤ *p* ≤ 0.001, *** *p* < 0.001 within groups; ^#^ *p* < 0.05 between groups. Scale bars: white, 1000 μm.

Mitochondrial mass was studied using MitoTracker Green (MTG), MitoTracker Deep Red (MTDR), and antibodies against membrane-associated proteins [51,52,53,54,55]. Live cell images of Ctrl1 and OPLL4 showed that fluorescence was increased in OPLL4 for MTG and MTDR (Figure 3a left). In addition, the fluorescence of MTG and MTDR was significantly higher in the OPLL group than in the Ctrl group (Figure 3a right). However, the expression of the mitochondrial outer membrane proteins TOM22 and VDAC was not statistically significant (Figure 3b). Mitochondrial mRNA expression for Complexes I~V was increased in the OPLL group as determined by RNA-seq (Supplementary Figure S6a–e). Mitochondrial mass was increased in the OPLL group according to mitochondrial probes and mRNA but not at the protein level.

The mitochondrial membrane potential (ΔΨ_M_) was studied using tetramethylrhodamine methyl ester (TMRM). Live cell images showed that OPLL4 exhibited higher fluorescence intensity for TMRM than Ctrl1 (Figure 3c, left) [2,56]. TMRM was increased in the OPLL group which implies that OPLL showed higher ΔΨ_M._ These membrane potentials were blocked by CCCP which is a mitochondria ETC uncoupling agent (Figure 3d) [19,20,21,22]. Mitochondrial ETC complexes were measured using antibodies against Complexes I to V. The protein staining intensity of Complexes III and IV was lower in the OPLL group than in the control group (Figure 3f, left), but the differences were not significant (Figure 3f, right).

Most icROS are derived from mitochondrial ROS (mtROS) in the form of superoxide (O_2_^•−^) [16,22]. Superoxide was stained with MitoSox and hydrogen peroxide was stained with 2′-7′-dichlorofluorescein diacetate (DCFDA). Staining was assessed using flow cytometry. These signals were stronger in OPLL4 than in Ctrl1 in live cell images (Figure 3g left) and stronger in the OPLL group than in the Ctrl group. The OPLL group showed higher MitoSox and DCFDA fluorescence than the Ctrl group (Figure 3g, right). These results showed that a higher membrane potential (ΔΨ_M_) in mitochondria resulted in greater mtROS production, which contributed to increased icROS in the OPLL group.

The levels of the ROS-scavenging enzymes GPX1, GPX4, SOD1, and SOD2 were decreased in the OPLL group (Figure 3h). The production of O_2_^•−^ was increased in the OPLL group, which was related to the fluorescence of the apoptosis indicator Annexin (Figure 3i). O_2_^•−^ was highly correlated with Annexin fluorescence (Pearson’s correlation method, r = 0.812), implying that apoptosis was increased according to O_2_^•−^ production, especially in the OPLL group (Figure 3j). Induction of autophagy by starvation of cells from 0 to 120 min decreased apoptosis in the Ctrl and OPLL groups (Figure 3k).

Altogether, these results showed that mitochondrial mass, ΔΨ_M_, mtROS production, and icROS were increased, whereas ROS scavenging proteins were decreased in the OPLL group. icROS, which act as pathological molecules, were associated with SLDC apoptosis in the OPLL group. These data and the downregulation of the lysosomal pathway in the OPLL group indicated by RNA-seq led to the hypothesis that autophagy might be decreased in the OPLL group.

### 2.5. Relationship between ROS and Ferritin in SLDC

To determine the effects of ROS in SLDCs, ferrous ammonium citrate (Fe), rotenone, and hydrogen peroxide (H_2_O_2_) were administered, and N-acetyl-L-cysteine (NAC), which is a ROS-scavenging molecule, was also studied. Rotenone is known to block mitochondrial complex I, which results in slightly increased levels of icROS from mitochondrial superoxide production [34]. Ferritin seems to have a contradictory ROS-producing or ROS-scavenging role other than its role in storing Fe [52,53,54,55,56].

**Figure 4:**
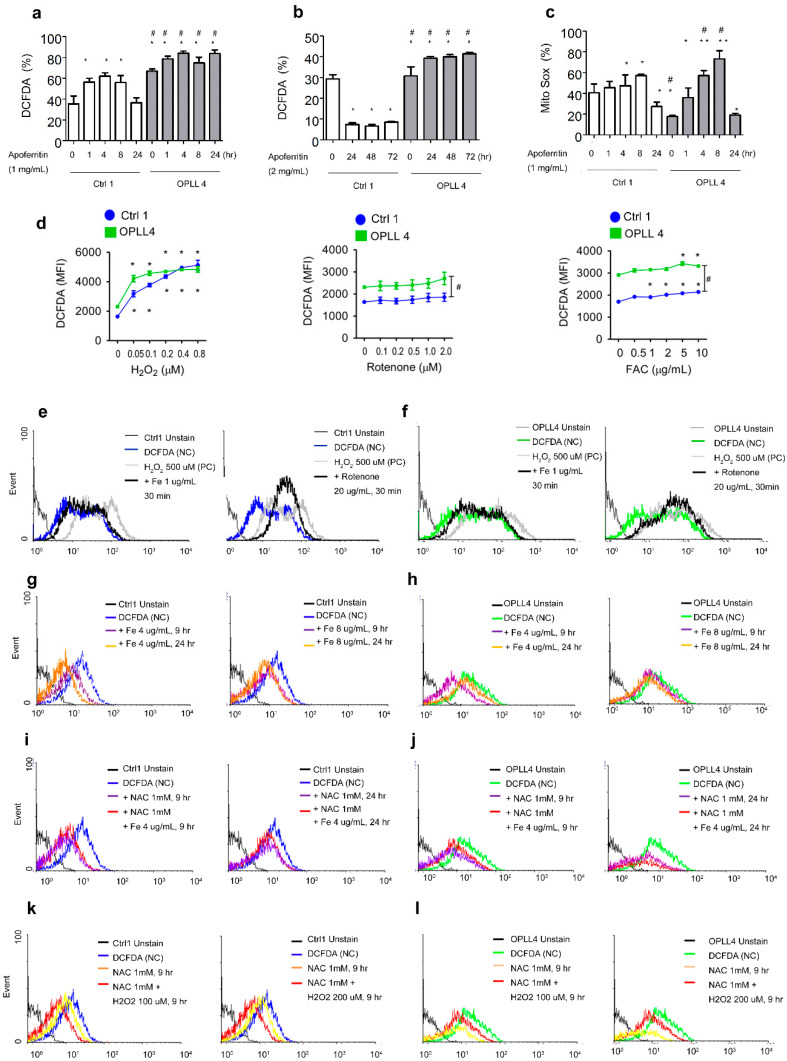
Assessment of intracellular reactive oxygen species (icROS) in spinal ligament-derived cells (SLDCs) from the control (Ctrl) and OPLL groups. (**a**) The effect of 1 mg/mL horse apoferritin on icROS status was studied for Ctrl1 and OPLL4 at 1, 4, 8, and 24 h. (**b**) The effect of 2 mg/mL apoferritin at 24, 48, and 72 h. (**c**) Effect of 1 mg/mL horse apoferritin on superoxide production. (**d**) ROS detection after addition of H_2_O_2_, rotenone, and ferric ammonium citrate (Fe). Saturation was reached after 0.05 µM H_2_O_2_ was added in Ctrl1, but there was a dose-dependent increase in DCFDA levels in OPLL4. Rotenone resulted in minimal and non-significant ROS production. Addition of Fe increased the ROS level after 4 µg/mL or 1 µg/mL was reached in Ctrl1 or OPLL4, respectively. In (**e**) Ctrl1 and (**f**) OPLL4 cells, ROS generation was induced by 1 μg/mL ferric ammonium citrate (Fe) and 20 μM rotenone, respectively. Rotenone, a mitochondrial NADH:ubiquinone reductase inhibitor, was used to treat the cells for 1 h. Flow cytometry was then used to measure ROS production after 25 μM DCFDA (%) staining for 30 min. In (**g**) Ctrl1 and (**h**) OPLL4, ROS were generated by incubation with 4 μg/mL Fe for 9 h and 24 h followed by 25 μM DCFDA (%) staining for 30 min. In (**i**) Ctrl1 and (**j**) OPLL4 cells, the effect of Fe on ROS production and the anti-ROS effect of N-acetyl-cysteine (NAC) were measured by flow cytometry using 25 μM DCFDA after 24 h of incubation. In (**k**) Ctrl1 and (**l**) OPLL4 cells, the effects of NAC and NAC along with H_2_O_2_ (100 µM, 200 µM) were analyzed. All the experiments were performed in triplicate, and throughout, the data are presented as the mean ± SEM. * *p* < 0.05, ** 0.001 ≤ *p* ≤ 0.001; within groups; ^#^ *p* < 0.05 between groups. Scale bars: white, 1000 μm.

Apoferritin induced ROS production until 8 h and repressed ROS from 8 h to 24 h. Over a longer time frame, apoferritin repressed ROS production from 24 h to 72 h (Figure 4a,b). Apoferritin induced superoxide (O_2_^•−^) production during the first 8 h in the Ctrl and OPLL groups, but a decrease in the O_2_^•−^ concentration was observed by 24 h (Figure 4c). The addition of H_2_O_2_ or Fe induced ROS production, but rotenone showed insignificant results (Figure 4d). The addition of 1 µg/mL Fe and 20 µM rotenone to Ctrl1 (Figure 4e) and OPLL4 (Figure 4f) for 30 min induced ROS production. In contrast, addition of 4 µg/mL Fe for 24 h especially repressed ROS production. As Fe induces ROS production via the Fenton reaction in a short time frame, the repression of ROS implies that Fe induced the production of ferritin, which acted as a ROS scavenger (Figure 4g) [57,58]. In OPLL4, treatment with 4 µg/mL Fe for 9 h resulted in repressed ROS formation (Figure 4h), whereas other concentration and time combinations showed no significant results. An elevated ROS concentration might accelerate the repressive reaction in OPLL.

The addition of NAC along with Fe showed no combined effect for Ctrl1 and OPLL4 (Figure 4i,j). The addition of NAC resulted in ROS clearance and, unexpectedly, the addition of NAC (1 mM) and H_2_O_2_ (100 µM) for 9 h repressed ROS accumulation to an even greater extent. These results indicated that H_2_O_2_ also induced anti-ROS protein or ferritin production. Increased H_2_O_2_ concentrations (200 µM) along with NAC showed decreased anti-ROS effects compared to 100 µM H_2_O_2_ (Supplementary Figure S7a,b). For Ctrl1, the addition of H_2_O_2_ repressed the ROS concentration in a dose- and time-dependent manner, whereas OPLL4 showed no difference (Supplementary Figure S7c–f).

Altogether, the results indicated that over a short time frame (<1 h), Fe and hydrogen peroxide (H_2_O_2_) promoted ROS production, whereas these molecules repressed ROS production over a longer frame (9 to 24 h). The addition of apoferritin induced ROS production by 9 h but repressed ROS production from 24 to 72 h. We hypothesized that Fe induced ferritin production and that H_2_O_2_ induced ferritin or anti-ROS protein production over a long time frame to result in ROS repression.

### 2.6. Effects of Fe, H_2_O_2_ and Apoferritin on Ferritin and ALP

**Figure 5:**
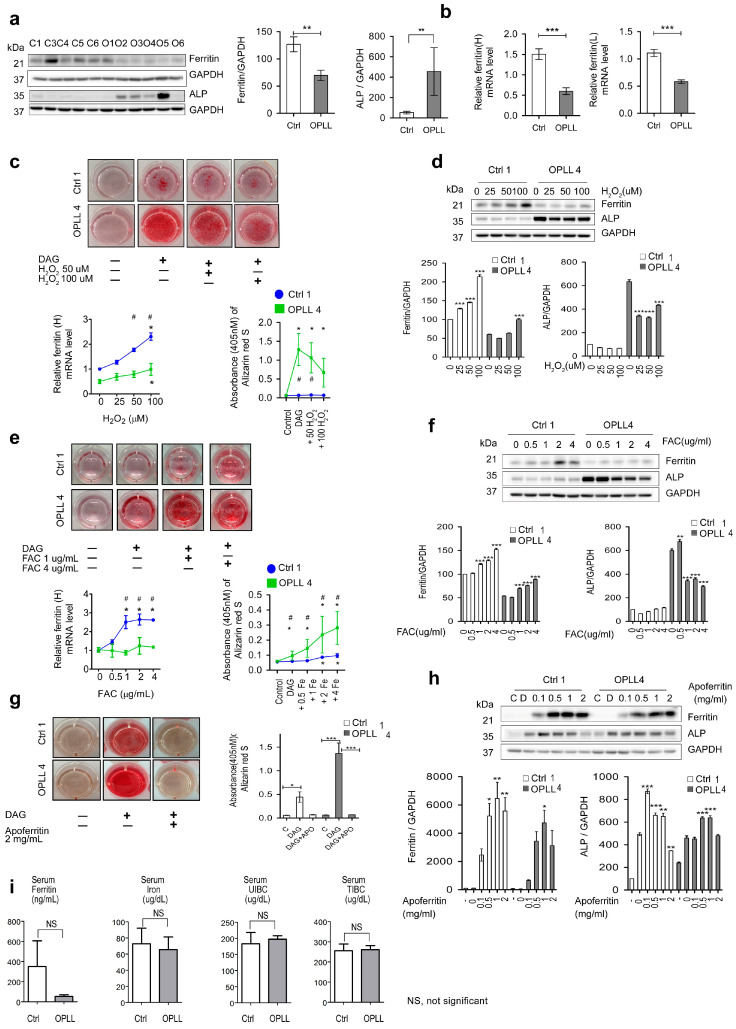
Baseline ferritin and ALP levels and induction of osteogenesis by H_2_O_2_, Fe, and apoferritin. (**a**) Western blot analysis of baseline ferritin heavy chain and ALP levels. The data are representative of triplicate tests. (**b**) Baseline ferritin light and heavy chain mRNA levels determined by qRT-PCR in the Ctrl and OPLL groups. (**c**, upper panel) Effect of H_2_O_2_ on osteogenic differentiation. The absorbance of ARS at 405 nM was measured for mineralization evaluation. (**c**, lower) Ferritin mRNA and ARS staining levels in cultured cells. (**d**) Western blot analysis of ferritin heavy chain and ALP levels induced by H_2_O_2_. (**e**, upper) Effect of ferritin ammonium citrate (Fe) on osteogenic differentiation. ARS staining was performed. (**e**, lower) Ferritin mRNA from cultured cells and ARS staining levels. (**f**) Western blot analysis of ferritin heavy chain and ALP levels under treatment with Fe from 0 to 4 µg/mL. (**g**) Effect of apoferritin (2 mg/mL) on osteogenic differentiation. ARS staining was evaluated to assess mineralization. (**h**) Western blot analysis of ferritin heavy chain and ALP levels under treatment with apoferritin from 0 to 2 mg/mL. (**i**) Serum ferritin, iron, total iron binding capacity (TIBC), and unbound iron binding capacity (UIBC) in serum from the four controls (Ctrl1, Ctrl4, Ctrl5, and Ctrl6) and OPLL groups 1 to 6 (n = 6). Throughout, the data are presented as the mean ± SEM. * *p* < 0.05, ** 0.001 < *p* ≤ 0.005, *** *p* < 0.001 within groups; ^#^ *p* < 0.05 between groups.

We investigated the enhanced osteogenic potential and the baseline study showed that the ferritin level was decreased but that the ALP level was increased in the OPLL group (Figure 5a) [58]. These results were also shown by the ferritin light and heavy chain mRNA expression levels by qRT-PCR (Figure 5b). Osteogenic differentiation resulted in increased ferritin or SOD2 levels by Day 14 in Ctrl1 (Supplementary Figure S8).

The addition of 25, 50, and 100 μM H_2_O_2_ decreased ossification in a dose-dependent manner (Figure 5c top). Ferritin mRNA expression was increased during the three weeks of osteogenesis (Figure 5c bottom). H_2_O_2_ addition resulted in ferritin induction in a dose-dependent manner, which led to ALP repression in Ctrl1 (Figure 5d). In the relative absence of ROS scavengers, H_2_O_2_ directly suppressed ALP in OPLL4 at 25 μM. Analysis of the effects of 0.5, 1, and 4 μg/mL Fe with DAG showed that 1.0 µg/mL Fe increased the osteogenesis level, whereas Fe at concentrations greater than 1.0 µg/mL repressed ossification (Figure 5e). Fe addition resulted in ferritin induction in a dose-dependent manner, which led to ALP repression in OPLL4 (Figure 5f). Ferritin was more strongly induced in Ctrl1, whereas ALP was more strongly repressed in OPLL4 by both H_2_O_2_ and ferritin. NAC at 1 mM for 24 h abrogated the ferritin level in Ctrl1, but the ALP level was stable, as the time frame was short (Supplementary Figure S9). The ALP levels in the culture supernatant were higher in the Ctrl group than in the OPLL group, and ALP seemed to be retained in the cytoplasm in the OPLL group (Supplementary Figure S10a–d).

Rotenone induced osteogenesis, only at a lethal dose (≥5 nM) but not at a physiological dose (Supplementary Figure S11a). Ferritin mRNA expression was induced by rotenone addition, but neither ferritin induction nor an ALP repression effect was noted (Supplementary Figure S11b).

The addition of 2.0 mg/dL apoferritin during osteogenic induction with DAG suppressed osteogenic differentiation in Ctrl1 and OPLL4 SLDCs (Figure 5g). Analysis after apoferritin protein (APO) addition from 0.1 to 2 mg/dL showed that apoferritin entered the cell in a dose-dependent manner. In Ctrl1, the ALP level was repressed by 0.5 mg/dL APO, whereas in OPLL4, the ALP protein level was repressed by 2.0 mg/dL APO (Figure 5h). In addition to ferritin, melatonin, α-tocopherol, and Fe enhanced osteogenic differentiation; these substances are known to possess anti-ROS functions. ROS producers inhibited osteogenic differentiation. Deferoxamine suppressed osteogenic differentiation by other mechanisms, including proliferation inhibition (Supplementary Figure S12).

To assess iron metabolism markers, serum iron, ferritin, UIBC, and TIBC were measured for the Ctrl (n = 4) and OPLL (n = 6) groups. Although the difference was not statistically significant, ferritin levels were decreased in the OPLL group (Figure 5i).

Altogether, the data indicated that, in the OPLL group, the baseline protein and gene expression levels of ferritin were decreased, whereas those of ALP were increased. H_2_O_2_ and Fe induced ferritin production, which resulted in repression of ALP. The decreased ferritin and increased ALP levels in the OPLL group seemed to be the cause of the increased osteogenic potential in the SLDCs. To verify these results, electronic medical records (EMRs) were studied to investigate these molecules in the peripheral blood.

### 2.7. Suggested Regulatory Mechanism

**Figure 6:**
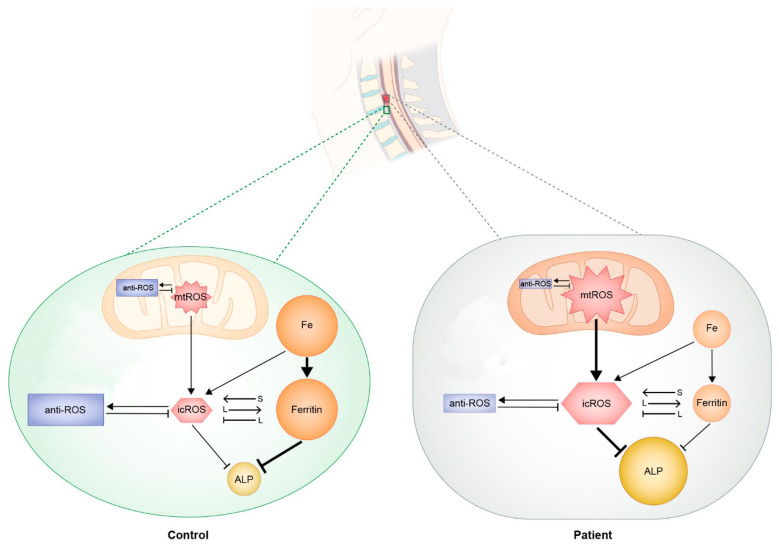
Proposed pathogenesis model for SLDCs in the Ctrl group and the ossification of the posterior longitudinal ligament (OPLL) group. The size of the circle or rectangle represents the relative amount of molecules. Reactive oxygen species (ROS) from mitochondria (mt) comprises most intracellular (ic) ROS. Iron (Fe) results in icROS production and induces ferritin. icROS induces ferritin over the long term (≥24 h). Ferritin results in icROS production over the short term (<24 h), and as ferritin is also an anti-ROS molecule, it represses icROS over the long term (≥24 h). Both icROS and ferritin repress ALP. In the OPLL group, autophagic flux and the ROS scavenging system are suppressed, which results in increased icROS. Fe and ferritin are scarce without repression of ALP in SLDCs from the OPLL group. S, short term; L, long term.

Based on the evidence, a regulatory mechanism between ROS (mostly from mitochondria) and ferritin was suggested (Figure 6). ROS or ferritin interacted in a context- or time-dependent manner within SLDCs from the Ctrl or OPLL group. ROS induced ferritin production over a long time frame or at high concentrations. Ferritin induced ROS production over a short time frame but repressed ROS production over a long time frame. Decreased ferritin and increased ALP levels within the SLDCs from the OPLL group were related to ossification. Increased mtROS levels from increased mitochondrial mass and decreased ROS-scavenging system activity seemed to damage cells, but these mechanisms paradoxically suppressed ALP production and induced ferritin production, which counterbalanced ossification. The lack of control of redox equilibrium seemed to damage cells on a small scale but in a constant manner, which eventually resulted in ossification.

Altogether, the data indicated that ROS and ferritin both suppressed ALP levels. Unlike the Ctrl group, the OPLL group showed scarce ferritin and markedly increased ALP in the baseline study. Thus, the mechanism was not fully functional in the OPLL group due to a lack of molecules.

## 3. Discussion

This study was performed by remnant tissues after surgery followed by recovering cells attached to the culture plate. The concept of mesenchymal stem/stromal cells (MSCs) has been proposed and renewed [59,60,61]. The MSCs showed distinct features including colony-forming (CFU) unit capability based on the origin of the tissue. The recovered SLDCs in this study showed Nestin + CD90 + CD105 + osteogenesis + chondrogenesis + adipogenesis CFU features. These common features were found in the Ctrl and OPLL groups but the degree of expression and differentiation capability were different between the Ctrl and OPLL groups. Further studies are required for elucidation of additional characteristics of these cells.

The regulatory mechanism between ROS and ferritin within SLDCs in the context of OPLL was identified in this study. Temporal regulation between ROS and ferritin was noted, as time was required for protein expression. ROS activity had a short time frame, whereas ferritin protein activity had a relatively long time frame. The temporal differences complicated the outcome of Fe or ROS addition. In our model, addition of Fe at 1 µg/mL or over a short time frame seemed to suppress ROS production during the incorporation of Fe into ferritin, which led to ALP expression; meanwhile, the addition of Fe at concentrations greater than 1 µg/mL or over a long time frame induced ferritin expression that led to suppression of ALP.

There was an age difference between the Ctrl and OPLL groups due to the availability of the tissue source. It has been reported that age-related changes in MSCs include loss differentiation potential, proliferation potential, increased senescent cell number, and loss of in vivo bone formation. In addition, the aging of MSCs favors adipogenic differentiation to osteogenic differentiation [62]. In this study, the OPLL group had enhanced proliferation potential and osteogenic and adipogenic differentiation. The older SLDCs from OPLL cells showed features congruent with younger MSCs. The old SLDCs showing younger features which might be associated with the pathophysiology of OPLL. Further studies are required for OPLL SLDCs that also favor adipogenic differentiation similar to the Ctrl group.

Autophagy plays a fundamental role in maintaining homeostasis in cells [27,28]. Autophagy is also related to redox equilibrium by means of specific mitochondrial degradation [29,30,31,32]. This process can alleviate the damage triggered by the accumulation of ROS and help restore redox balance [31,32]. In addition, ROS are related to osteogenic or adipogenic differentiation in mesenchymal stem/stromal cells [12,13,14,15]. Although there is limited evidence, autophagy is reported to be associated with ossification in OPLL patients. As autophagy is related to bone formation, ossification might be closely related to autophagy and might play an important role in ossification [63].

MitoSox levels were increased in the OPLL group. In older subjects, mitochondria showed lowered oxidative capacity, reduced oxidative phosphorylation, increased ROS production, lowered antioxidant defense and mitochondria density, dynamics were gradually decreased, and mitochondria-mediated apoptosis is increased [64,65]. In this study, mitochondrial mass and production of mitochondrial superoxide measured by MitoSox were increased. We thought that the age factor and decreased SOD2 protein levels that could defend against superoxide were associated with increased MitoSox levels which is equivalent to ROS. 

Although the mechanism underlying OPLL has been suggested, there might be an additional mechanism that leads to enhancement or attenuation of the osteogenic potential. In this study, our findings suggested that ferritin augmentation or induction might suppress ALP activation, which might alleviate the progression of abnormal ossification. Supplementation with a sufficient amount of iron can induce ferritin, which can repress ALP. Additional studies with more patients and clinical trials are required for validation of possible medical treatments.

MSCs have been considered a therapeutic source for the treatment of tissue regeneration. The osteogenic potential of MSCs could be utilized as a biomaterial for bone formation supplements [66,67,68]. Biomechanical stress and the addition of chemical agents induced bone formation for MSCs, which could be applied during culture. As OPLL SDLCs showed lower ferritin levels, inhibition or mitigation of ferritin might enhance the osteogenic potential in MSCs. It was reported that injury of the nucleus pulposus results in degeneration and loss of integrity [66,67,68]. The characteristics of OPLL might be applied to potentiate annulus fibrosis repair or meet biomechanical requirements for stabilization of the nucleus pulposus.

A limitation of this study is that the increased mitochondrial mass and ALP levels and the decreased ferritin levels were not explained. Aging might be a prominent factor associated with increased mitochondrial activity or increased production of ROS. The age difference between the Ctrl and OPLL groups was because operable cervical herniated nucleus pulposus patients were younger than the OPLL patients. Considering that OPLL is an age-dependent chronic degenerative disease, the study results could reflect age related features or biology, which might be closely related to pathophysiology. The model proposed in this study only describes sufficient conditions for increasing or decreasing osteogenic potential. Apoptosis was increased in SLDCs from OPLL patients, and the dynamic crosstalk between autophagy and apoptosis requires further study.

## 4. Materials and Methods

### 4.1. Patients

This study was a single-center, prospective study that was performed according to the ethical standards of the Declaration of Helsinki and approved by the institutional review board of Incheon St. Mary’s Hospital and Seoul St. Mary’s Hospital. A total of five patients diagnosed with cervical herniated nucleus pulposus (Control, Ctrl) and six patients diagnosed with ossification of the posterior longitudinal ligament (OPLL) scheduled for decompression surgery or laminectomy were enrolled from January 2014 to December 2014. The demographics, diagnosis, and surgical treatments of the patients are listed in Table 1.

### 4.2. Cell Culture

Surgical tissues were delivered from the operating room in an aseptic 20 mL glass tube in phosphate-buffered saline (PBS). SLDCs from the five control and six OPLL patients that adhered to the bottom of the culture plate were isolated. The tissue was cultured, harvested, and cryopreserved according to a previous method with modification [69,70]. SLDCs from passages 3 to 6 were cultured and harvested throughout the experiments. 

### 4.3. In Vitro Differentiation

The SLDCs were differentiated into three lineages as previously described with modification [69,70]. Osteogenic differentiation was performed using 10 nM dexamethasone (D8893; Merck Millipore, Burlington, MA, USA), 250 μM ascorbic acid (A4544; Sigma Aldrich, St. Louis, MO, USA), and 10 mM β-glycerophosphate (G9422; Sigma Aldrich). Adipogenic differentiation was performed using 1 μM dexamethasone, 10 μg/mL insulin (Humalog; Novornodisk, Bagsvaerd, Denmark), 200 μM indomethacin (17378; Sigma Aldrich), and 500 μM IBMX (I5879, Sigma Aldrich). The chondrogenic medium consisted of 100 nM dexamethasone, 50 μM ascorbic acid, 10 ng/mL TGF-β3 (8791; Merck Millipore), and 10 μM insulin. For osteogenesis and adipogenesis, 1.5 × 10^4^ cells/well were inoculated into 6-well culture plates and cultured for up to 21 days in a 37 °C incubator. Alizarin red S (A5533; Merck Millipore) staining was performed to confirm mineral deposition, and Oil red O (O0625; Merck Millipore) staining was performed to analyze lipid droplets. For chondrogenesis, after 6 weeks of culture, the cell pellets were stained with Alcian blue (A9186; Merck Millipore) and H&E. For quantification of mineralization, the wells were washed and stained with Alizarin Red S. The wells were dried and 10% acetic acid was added for 30 min with shaking. The harvested cells were incubated in a heat block at 85 °C for 10 min. After centrifugation, 10% ammonium hydroxide was added and the absorbance was measured at 405 nm [69,70,71]. The standard culture medium consisted of DMEM (12-60F; Lonza Bioscience, Morrisville, NC, USA), 20% fetal bovine serum (16000-044; Gibco ThermoFisher Scientific, Waltham, MA, USA), 1% penicillin–streptomycin (15140-122; Gibco), and Glutamax (35050-061; Gibco). For Oil Red O staining, the cells were fixed followed by the addition of 60% isopropranolol for 5 min and 2 mL of Oil Red O solution was added for 5 min. After washing, the plates were analyzed by phase contrast microscopy [69,70,71].

### 4.4. Cell Proliferation Assay

For the SLDCs from the five control and sox OPLL patients, cell proliferation was measured with a Cell Counting Kit-8 (CK04-11; Dojindo Laboratories, Kumamoto, Japan). Eight hundred cells in 100 mL of medium were inoculated into a 96-well culture plate. The medium consisted of DMEM (12-604F; Lonza), 10% FBS, and antibiotics. The cells were stained with 10 μL of CCK-8 reagent after 2 h of incubation. The absorbance was measured by spectrometry at 450 nm from Day 0 to Day 14.

### 4.5. Real-Time Quantitative Polymerase Chain Reaction (qRT-PCR) and Conventional PCR

RNA was extracted, and cDNA was prepared (Roche Diagnostics, Basel, Switzerland). Single gene expression was measured by RT-PCR using SYBR Green reagent (RR430A; Takara Bio Inc., Kusatsu, Japan). PCR was performed on an ABI 7500 System (ThermoFisher). The relative mRNA expression level was calculated with the delta-delta CT method with GAPDH as an internal control. The Forward and Reverse primers used are listed in Table 2.

### 4.6. Flow Cytometric Analysis

Flow cytometric analysis was performed with a FACSCanto II and FACSDiva software (BD Biosciences, San Jose, CA, USA). The SLDCs were cultured in 100 mm petri dishes and harvested at 70% confluency. The cells in PBS were analyzed at a concentration of 1 × 10^6^ cells/mL. For investigation of stemness, cell surface expression markers were used in 10 μL [72], and 10,000 events were acquired for analysis. All flow cytometric reagents are from BD Bioscience: HLA-DR-FITC (BD555558); IgG2A-FITC (BD555571); CD79A-PE (BD 555935); CD11b-PE (BD561355); CD45-PE (BD555483); CD90-PE (BD555596); CD73-PE (BD550257); CD105-PE (BD560839); CD146-PE (BD550315); and Annexin V apoptosis detection kit (BD 556547). 

For ROS assessment, the cells were incubated with DCFDA (Sigma, D6883, 25 μM). For mitochondrial superoxide, mitochondrial number, and mitochondrial membrane potential assessments, MitoSox (M36008, 5 μM; ThermoFisher), MitoTracker Green (M7514, 200 nM; ThermoFisher), MitoTracker Deep Red (M22426, 500 nM; Thermo Fisher), and TMRM (T9281, 200 nM; Thermo Fisher) were used [73,74]. For MitoSox staining, HBSS was used with a 30 min incubation at 37 °C. The cells were washed twice with PBS and harvested at 70% confluency. For the apoptosis analysis, Annexin V FITC was measured in the Ctrl and OPLL groups using flow cytometry.

### 4.7. ALP Concentration Assessment

Alkaline phosphatase was measured using the supernatant from the culture medium on Days 3, 7, 10, and 14, and using the supernatant from the culture medium up to Day 21. The supernatant was measured using an AU5800 automated chemistry analyzer (Beckman Coulter Diagnostics, Brea, CA, USA).

### 4.8. Western Blot Analysis

Linearity graphs, equal number of cells, loading justified amount of samples, and normalization are some of the important features for quality control of Western blot for quantification, which was performed in this study [75,76]. The cultured cells were lysed in RIPA buffer (R2002; Biosesang, Seongnam, Korea) and incubated on ice for 10 min. The protein was quantified with a BCA assay kit (23227; ThermoFisher). Loading buffer (Bio-Rad, Hercules, CA, USA) and 10 μg of protein were mixed and then loaded onto a 12.5% acrylamide gel prepared from a 30% acrylamide/Bis solution (1610158; Bio-Rad). Tris–glycine buffer (10×) and 10× CAPS buffer were used as the running and transfer buffers, respectively (TR2028; Biosesang). Electrophoresis was performed at 80–120 mA for 24 h. Blocking was performed using 5% skim milk overnight at 4 °C. Transfer to a nitrocellulose membrane was performed at 200 mA for 1 h. The primary and secondary antibodies are listed in the reagents. The primary and secondary antibodies were used at dilutions of 1:1000 and 1:3000 and incubated for 2 h and 1 h, respectively. Washing was performed three to four times using TBST (TR2007; Biosesang) with 5% skim milk. The following antibodies (ab) were used for Western blot analysis: anti-ferritin ab (ab75972; Abcam Inc, Boston, MA, USA); anti-ALP ab (Abcam, ab354); anti-VDAC ab (Abcam, ab34726); anti-TOM22 ab (Abcam, ab57523); anti-mitochondrial complex 1~5 ab cocktail (Abcam, 110413); anti-nestin ab (ThermoFisher, MA1-110); anti-GPX1 ab (3286; Cell Signaling Technologies, Danvers, MA, USA); anti-GPX4 ab (166120; Abcam); anti-SOD1 ab (ab16831; Abcam); anti-SOD2 ab (13141; Cell signaling); anti-tubulin ab (ab7291; Abcam); anti-rabbit IgG ab (7074s; Cell signaling); anti-mouse IgG ab (7076s; Cell Signaling); and goat anti-mouse IgG ab (35502; Thermo Fisher).

### 4.9. Live Cell Imaging

For live cell imaging, 1.5 × 10^4^ cells/well were inoculated into 4-well chamber slides for 2 days in a 37 °C incubator with 5% CO_2_. After fixation for 15 min with 4% paraformaldehyde in PBS, the cells were permeabilized with 0.5% Triton X-100 in PBS for 15 min. Blocking was performed using 3% BSA in PBS. The cells were incubated with antibodies in 3% BSA for 3 h. The cells were incubated with secondary antibodies for 1 h at room temperature in 3% BSA in PBS. DAPI was used for nuclear staining. To stain mitochondria using MTG (200 nM), MTDR (500 nM), MitoSox (5 µM), DCFDA (25 μM), and TMRM (200 nM), the fixation and blocking steps were omitted, and incubated was performed at room temperature for 30 min.

### 4.10. RNA-Seq Analysis

RNA was extracted after 3 days of SLDC culture. RNA sequencing was performed for all SLDCs from the five control and six OPLL patients as previously described with modification [43,46]. cDNA synthesis, library preparation, and RNA sequencing on an Illumina HiSeq 2000 were performed by Macrogen (Seoul, Korea) [43,46]. Approximately 40 million 150 bp paired-end reads were analyzed. The fragments per kilobase of exon per million mapped fragments (FPKM) values and raw read count data were obtained for further analysis. The statistical analysis was performed using R software, and related package-driven analyses and graphical work were performed using ggplot2.

### 4.11. Statistical Analysis

The parameters are expressed as the mean ± SD for continuous variables and the mean ± SEM for countable variables. The Mann–Whitney U test and Kruskal–Wallis test were used for comparing two groups and multiple groups, respectively. For statistical analysis, *p* < 0.05 was considered to indicate a significant result. All the statistical analysis was performed suing R software v 3.4.4 (Free Software Foundation, Boston, MA, USA).

## Figures and Tables

**Table 1:** Clinical characteristics of Control (Ctrl) and OPLL patients enrolled in study.

No	Age	Sex	Involved Spine	Treatment
Ctrl1	39	Female	C6,7	Total disc replacement
Ctrl3	36	Male	C5,6	Total disc replacement
Ctrl4	32	Female	C5,6	Total disc replacement
Ctrl5	46	Female	C4,5, C5,6	Anterior cervical interbody fusion
Ctrl6	62	Female	C5,6	Anterior cervical interbody fusion
OPLL1	65	Female	C4,5,6,7	Laminoplasty
OPLL2	61	Male	C4,5,6,7	Laminoplasty
OPLL3	66	Female	C2,3,4,5,6,7	Laminoplasty, Partial hemilaminectomy
OPLL4	61	Male	C4,5,6	Laminoplasty
OPLL5	52	Female	C2,3,4,5,6,7	Laminoplasty, Partial hemilaminectomy
OPLL6	67	Male	C2,3,4,5,6,7	Laminoplasty

Control, cervical herniated nucleus pulposus patients; OPLL, ossification of posterior longitudinal ligament; C, cervical.

**Table 2:** Primers used for differentiation and stemness genes.

Primer		Sequence	Amplicon (bp)
Differentiation			
*GAPDH*	F	5′-GGA GTC CAC TGG CGT CTT CA-3′	123
	R	5′-TGG TTC ACA CCC ATG ACG AA-3′	
*BMP2*	F	5′-TTCGGCCTGAAACAGAGAC-3′	199
	R	5′-TCCCACTCGTTTCTGGTAGT-3′	
*RUNX2*	F	5′-AGATGACGTCCCCGTCCATC-3′	215
	R	5′-TGAAATGCTTGGGAACTGCC-3′	
*Osteocalcin*	F	5′-CCTCACAC CCTCGCCCATT-3′	117
	R	5′-CCCTCCTGCTTGGACACAAA-3′	
*ALP*	F	5′-CCCACCTACAGCATGTCCTA-3′	195
	R	5′-AATTCTGCCTCCTTCCACCA-3′	
*PPARG*	F	5′-TGACCCAGAAAGCGATTCCT-3′	100
	R	5′-AAA GTT GGT GGG CCA GAA TG-3′	
*SOX9*	F	5′-GTGCTCAAAGGCTACGACTG-3′	200
	R	5′-AGAAGTCTCCAGAGCTTGCC-3′	
*COL2A1*	F	5′-ACATGCCGTGACTTGAGACTCA-3′	152
	R	5′-GGGATGTTTTCAGGTTGGGC-3′	
*MSX1*	F	5′-CTCTCAAGCTGCCAGAAGAT-3′	150
	R	5′-GTTCGTCTTGTGTTTGCGGA-3′	
*FTH*	F	5′-GCTGAATGCAATGGAGTGTG-3′	335
	R	5′-CAGGGTGTGCTTGTCAAAGA-3′	
*FTL*	F	5′-AGAAGATGGGTGACCACCTG-3′	85
	R	5′-TGGTCCAAGGCTTGTTAGGA-3′	
Stemness			
*GAPDH*	F	5′-ACAACTTTGGTATCGTGGAA-3′	456
	R	5-AAATTCGTTGTCATACCAGG-3′	
*OCT4*	F	5-CGTGAAGCTGGAGAAGGAGAAGCTG-3′	245
	R	5′-CAAGGGCCGCAGCTCACACATGTT-3′	
*SOX2*	F	5′-GCCGAGTGGAAACTTTTGTC-3′	264
	R	5′-GTTCATGTGCGCGTAACTGT-3′	
*NANOG*	F	5′-AGAAGGCCTCAGCACCTAC-3′	250
	R	5′-GGCCTGATTGTTCCAGGATT-3′	

Abbreviation: F, forward; R, reverse; bp, base pair.

## Data Availability

Data is contained within the article or Supplementary Materials.

## Supplementary Materials

The following supporting information can be downloaded at: https://www.mdpi.com/article/10.3390/ijms24032872/s1.

## References

[1] Inamasu J., Guiot B., Sachs D. (2006). Ossification of the posterior longitudinal ligament: An update on its biology, epidemiology, and natural history. Neurosurgery.

[2] Matunaga S., Sakou T. (2012). Ossification of the posterior longitudinal ligament of the cervical spine. Spine.

[3] Sohn S., Chung C.K., Yun T.J., Sohn C.H. (2014). Epidemiological survey of ossification of the posterior longitudinal ligament in an adult Korean population: Three-dimensional computed tomographic observation of 3,240 Cases. Calcif. Tissue Int..

[4] Kim T., Bae K., Uhm W., Kim T., Joo K., Jun J. (2008). Prevalence of ossification of the posterior longitudinal ligament of the cervical spine. Jt. Bone Spine.

[5] Kwon S.Y., Shin J.J., Lee J.H., Cho W.H. (2015). Prognostic factors for surgical outcome in spinal cord injury associated with ossification of the posterior longitudinal ligament (OPLL). J. Orthop. Surg. Res..

[6] Head J., Rymarczuk G., Stricsek G., Velagapudi L., Maulucci C., Hoelscher C., Harrop J. (2019). Ossification of the posterior longitudinal ligament: Surgical approaches and associated complications. Neurospine.

[7] Huang W., Yang S., Shao J., Li Y.P. (2007). Signaling and transcriptional regulation in osteoblast commitment and differentiation. Front. Biosci..

[8] Stapleton C.J., Pham M.H., Attenello F.J., Hsieh P.C. (2011). Ossification of the posterior longitudinal ligament: Genetics and pathophysiology. Neurosurg. Focus..

[9] Karasugi T., Nakajima M., Ikari K., Tsuji T., Matsumoto M., Chiba K., Uchida K., Kawaguchi Y., Mizuta H., Ogata N. (2013). A genome-wide sib-pair linkage analysis of ossification of the posterior longitudinal ligament of the spine. J. Bone Miner. Metab..

[10] Jekarl D.W., Paek C., An Y.J., Kim Y.J., Kim M., Kim Y., Lee J., Sung C.H. (2013). TGFBR2 gene polymorphism is associated with ossification of the posterior longitudinal ligament. J. Clin. Neurosci..

[11] Nakajima M., Takahashi A., Tsuji T., Karasugi T., Baba H., Uchida K., Kawabata S., Okawa A., Shindo S., Takeuchi K. (2014). A genome-wide association study identifies susceptibility loci for ossification of the posterior longitudinal ligament of the spine. Nat. Genet..

[12] Harada Y., Furukawa K., Asari T., Chin S., Ono A., Tanaka T., Mizukami H., Murakami M., Yagihashi S., Motomura S. (2014). Osteogenic lineage commitment of mesenchymal stem cells from patients with ossification of the posterior longitudinal ligament. Biochem. Biophys. Res. Commun..

[13] Kudo H., Furukawa K.-I., Yokoyama T., Ono A., Numasawa T., Wada K., Tanaka S., Asari T., Ueyama K., Motomura S. (2011). Genetic Differences in the Osteogenic Differentiation Potency According to the Classification of Ossification of the Posterior Longitudinal Ligament of the Cervical Spine. Spine.

[14] Atashi F., Modarressi A., Pepper M. (2015). The role of reactive oxygen species in mesenchymal stem cell adipogenic and osteogenic differentiation: A review. Stem Cells Dev..

[15] Imhoff B.R., Hansen H.M. (2011). Differentiation redox potential profiles during adipogenesis and osteogenesis. Cell Mol. Biol. Lett..

[16] Lee K.J., Clegg P.D., Comerford E.J., Canty-Laird E.G. (2017). Ligament-derived stem cells: Identification, characterization and therapeutic application. Stem Cells Int..

[17] Kristjansson B., Limthongkul W., Yingsakmongkol W., Thantiworasit P., Jirathanathornnukul N., Honsawek S. (2016). Isolation and characterization of human mesenchymal stem cells from facet joints and interspinous ligaments. Spine.

[18] Bianco P., Cao X., Frenette P.S., Mao J.J., Robey P.G., Simmons P.J., Wang C. (2013). The meaning, the sense and the significance: Translating the science of mesenchymal stem cells into medicine. Nat. Med..

[19] Sacchetti B., Funari A., Remoli C., Giannicola G., Kogler G., Liedtke S., Cossu G., Serafini M., Sampaolesi M., Tagliafico E. (2016). No Identical “Mesenchymal Stem Cells” at Different Times and Sites: Human Committed Progenitors of Distinct Origin and Differentiation Potential Are Incorporated as Adventitial Cells in Microvessels. Stem Cell Rep..

[20] Ray P.D., Huang B.W., Tsuji Y. (2012). Reactive oxygen species (ROS) homeostasis and redox regulation in cellular signaling. Cell Signal.

[21] Murphy M.P. (2009). How mitochondria produce reactive oxygen species. Biochem. J..

[22] Korshunov S.S., Skulachev V.P., Starkov A.A. (1997). High protonic potential actuates a mechanism of production of reactive oxygen species in mitochondria. FEBS Lett..

[23] Zorov D.B., Juhaaszova M., Sollott S.J. (2014). Mitochondrial reactive oxygen species (ROS) and ROS-induced ROS release. Physiol. Rev..

[24] Dunn J.D., Alvarez L.A., Zhang X., Soldati T. (2015). Reactive oxygen species and mitochondria: A nexus of cellular homeostasis. Redox. Biol..

[25] Schulz E., Wenzel P., Munzel T., Daiber A. (2014). Mitochondrial redox signaling: Interaction of mitochondrial reactive oxygen species with other source of oxidative stress. Antioxid. Redox Signal.

[26] Kane M.S., Paris A., Codron P., Cassereau J., Procaccio V., Lenaers G., Reynier P., Chevrollier A. (2018). Current mechanistic insights into the CCCP-induced cell survival response. Biochem. Pharmacol..

[27] Kwon K., Viollet B., Yoo O. (2011). CCCP induces autophagy in an AMPK-independent manner. Biochem. Biophys. Res. Commun..

[28] Bresgen N., Eckl P. (2015). Oxidative stress and the homeodynamics of iron metabolism. Biomolecules.

[29] Dikic I., Elazar Z. (2018). Mechanism and medical implications of mammalian autophagy. Nat. Rev. Mol. Cell Biol..

[30] Wang Y., Nartiss Y., Steipe B., McQuibban A., Kim P.K. (2012). ROS-induced mitochondrial depolarization initiates RARK2/PARKIN-dependent mitochondrial degradation by autophagy. Autphagy.

[31] Levine B., Kroemer G. (2019). Biological functions of autophagy genes: A disease perspective. Cell.

[32] Klionsky D.J., Abdel-Aziz A.K., Abdelfatah S., Abdellatif M., Abdoli A., Abel S., Abeliovich H., Abildgaard M.H., Abudu Y.P., Acevedo-Arozena A. (2021). Guidelines for the use and interpretation of assays for monitoring autophagy (4th edition). Autophagy.

[33] Filomeni G., De Zio D., Cecconi F. (2015). Oxidative stress and autophagy: The clash between damage and metabolic needs. Cell Death Differ..

[34] Frank M., Duvezin-Caubet S., Koob S., Occhipinti A., Jagasia R., Petcherski A., Ruonala M.O., Priault M., Salin B., Reichert A.S. (2012). Mitophagy is triggered by mild oxidative stress in a mitochondrial fission dependent manner. Biochim. Biophys. Acta (BBA) Mol. Cell Res..

[35] Youle R.J., Narendra D.P. (2011). Mechanism of mitophagy. Nat. Rev. Mol. Cell Biol..

[36] White E. (2015). Autophagy and p53. Cold Spring Harb. Perspect. Med..

[37] Bou-Abdallah F. (2018). The iron redox and hydrolysis chemistry of the ferritins. Biochim. Biophys. Acta.

[38] Zhao G., Arosio P., Chasteen N.D. (2006). Iron(II) and hydrogen peroxide detoxification by human H-chain ferritin. An EPR spin-trapping study. Biochemistry.

[39] Tori F.M., Tori S.V. (2012). Regulation of ferritin genes and protein. Blood.

[40] Theil E.C. (2013). Ferritin: The protein nanocage and iron biomineral in health and in disease. Inorg. Chem..

[41] Zarjou A., Jeney V., Arosio P., Poli M., Zavaczki E., Balla G., Balla J. (2010). Ferritin ferroxidase activity: A potent inhibitor of osteogenesis. J. Bone Miner. Res..

[42] Zarjou A., Jeney V., Arosio P., Poli M., Antal-Szalmás P., Agarwal A., Balla G., Balla J. (2009). Ferritin prevents calcification and osteoblastic differentiation of vascular smooth muscle cells. J. Am. Soc. Nephrol..

[43] Wang Z., Gerstein S.M. (2009). RNA-Seq: A revolutionary tool for transcriptomics. Nat. Rev. Genet..

[44] Stark R., Grzelak M., Hadfield J. (2019). RNA sequencing: The teenage years. Nat. Rev. Genet..

[45] Bolger A.M., Lohse M., Usadel B. (2014). Trimmomatic: A flexible trimmer for illumina sequence data. Bioinformatics.

[46] Seo J.-S., Rhie A., Kim J., Lee S., Sohn M.-H., Kim C.-U., Hastie A., Cao H., Yun J.-Y., Kim J. (2016). De novo assembly and phasing of a Korean human genome. Nature.

[47] Robinson M.D., McCarthy D.J., Smyth G.K. (2010). edgeR: A Bioconductor package for differential expression analysis of digital gene expression data. Bioinformatics.

[48] Ritchie M.E., Phipson B., Wu D., Hu Y., Law C.W., Shi W., Smyth G.K. (2015). limma powers differential expression analyses for RNA-sequencing and microarray studies. Nucleic Acids Res..

[49] Huang D.W., Sherman B.T., Lempicki R.A. (2009). Systemic and integrative analysis of large gene lists using DAVID bioinformatics resources. Nat. Protoc..

[50] Luo W., Friedman M.S., Shedden K., Hankenson K.D., Woolf P.J. (2009). GAGE: Generally applicable gene set enrichment for pathway analysis. BMC Bioinform..

[51] Mandal S., Lindgren A.G., Srivastava A.S., Clark A.T., Banderjee U. (2010). Mitochondrial function controls proliferation and early differentiation potential of embryonic stem cells. Stem Cells.

[52] Sivandzade F., Bhalerao A., Cucullo L. (2019). Analysis of the mitochondrial membrane potential using the cationic JC-1 dyes as a sensitive fluorescent probe. Bio Protoc..

[53] Monteiro L.L., Davanzo G.G., de Aguiar C.F., Moraes-Vieria P.M.M. (2020). Using flow cytometry for mitochondrial assays. MethodsX.

[54] Perry S.W., Norman J.P., Barbieri J., Brown E.B., Gelbard H.A. (2011). Mitochondrial membrane potential probes and the proton gradient: A practical usage guide. Biotechniques.

[55] Dolman N.J., Chambers K.M., Mandavilli B., Batchelor R.H., Janes M.J. (2013). Tools and techniques to measure mitophagy using fluorescence microscopy. Autophagy.

[56] Kohen R., Nyska A. (2002). Oxidation of biological system: Oxidative stress phenomena, antioxidants, redox reactions, and methods for their quantification. Tox. Pathol..

[57] Sies H., Jones D.P. (2020). Reactive oxygen species (ROS) as pleiotropic physiological signalling agents. Nat. Rev. Mol. Cell Biol..

[58] Rader B.A. (2017). Alkaline phosphatase, an unconventional immune protein. Front. Immunol..

[59] Sacchetti B., Augello A., Bari D.C. (2010). The regulation of differentiation in mesenchymal stem cells. Hum. Gene Ther..

[60] Pittenger M.F., Discher D.E., Peault B.M., Phinney D.G., Hare J.M., Caplan A.I. (2019). Mesencymal stem cell perspective: Cell biology to clinical progress. Regen. Med..

[61] Anthony B.A., Link D.C. (2014). Regulation of hematopoietic stem cells by bone marrow stromal cells. Trends Immunol..

[62] Kim M., Kim C., Choi Y.S., Kim M., Park C., Suh Y. (2012). Age-related alterations in mesenchymal stem cells related to shift in differentiation from osteogenic to adipogenic potential: Implication to age-associated bone diseases and defects. Mech. Ageing Dev..

[63] Yang Y., Lin Z., Chen J., Ding S., Mao W., Shi S., Liang B. (2020). Autophagy in spinal ligament fibroblasts: Evidence and possible implications for ossification of the posterior longitudinal ligament. J. Orthop. Surg. Res..

[64] Palmer C.S., Osellame L.D., Stojanovski D., Ryan M.T. (2011). The regulation of mitochondrial morphology: Intricate mechanisms and dynamic machinery. Cell. Signal..

[65] Chistiakov D.A., Sobenin I.A., Revin V.V., Orekhove A.N., Bobryshev Y.V. (2014). New insights into vascular aging: Emerging role of mitochondria function. Biomed. Res. Int..

[66] Bryniarska N., Kubiak A., Labedz-Maslowska A., Zuba-Surma E. (2019). Impact of developmental origin, niche mechanics and oxygen availability on osteogenic differentiation capacity of mesenchymal stem/stromal cells. Acta Biochim. Pol..

[67] Iatridis J.C., Nicoll S.B., Michalek A.J., Walter B.A., Gupta M.S. (2013). Role of biomechanics in intervertebral disc degeneration and regenerative therapies: What needs repairing in the disc and what are promising biomaterials for its repair?. Spine J..

[68] Inoue Z., Orias A.A.E. (2011). Biomechanics of intervertebral disk degeneration. Orthop. Clin. N. Am..

[69] Lee S., Park B.-J., Kim J.Y., Jekarl D., Choi H.Y., Lee S.Y., Kim M., Kim Y., Park M.-S. (2015). The effect of fibroblast growth factor on distinct differentiation potential of cord blood–derived unrestricted somatic stem cells and Wharton’s jelly–derived mesenchymal stem/stromal cells. Cytotherapy.

[70] Kwon A., Kim Y., Kim M., Kim J., Choi H., Jekarl D.W., Lee S., Kim J.M., Shin J.-C., Park I.Y. (2016). Tissue-specific Differentiation Potency of Mesenchymal Stromal Cells from Perinatal Tissues. Sci. Rep..

[71] Gregory C.A., Gunn W.G., Peister A., Prockop D. (2004). An Alizarin red-based assay of mineralization by adherent cells in culture: Comparison with cetylpyridinium choride extraction. Anal. Biochem..

[72] Scott M.A., Nguyen V.T., Levi B., James A.W. (2011). Current methods of adipogenic differentiation of mesenchymal stem cells. Stem Cells Dev..

[73] Dominici M., Le Blanc K., Mueller I., Slaper-Cortenbach I., Marini F., Krause D., Deans R., Keating A., Prockop D., Horwitz E. (2006). Minimal criteria for defining multipotent mesenchymal stromal cells. The International Society for Cellular Therapy position statement. Cytotherapy.

[74] Mukhopadhyay P., Rajesh M., Haskó G., Hawkins B.J., Madesh M., Pacher P. (2007). Simultaneous detection of apoptosis and mitochondrial superoxide production in live cells by flow cytometry and confocal microscopy. Nat. Protoc..

[75] Gassmann M., Grenacher B., Rohde B., Vogel J. (2009). Quantifying western blots: Pitfalls of densitometry. Electrophoresis.

[76] Butler T.A., Paul J.W., Chan E., Smith R., Tolosa J.M. (2019). Misleading westerns: Common quantification mistakes in Western blot densitometry and proposed corrective measures. Biomed. Res. Int..

