# Supplementary material for: Regulatory Mechanism between Ferritin and Mitochondrial Reactive Oxygen Species in Spinal Ligament-Derived Cells from Ossification of Posterior Longitudinal Ligament Patient
Source: Int J Mol Sci. 2023 Feb 2;24(3):2872. doi: 10.3390/ijms24032872 (PMC9917908; doi:10.3390/ijms24032872)
Supplement: Supplementary file 1 [file ijms-24-02872-s001.zip › ijms-2175239-supplementary.pdf]

Supplementary Information for

Regulatory Mechanism between Ferritin and Mitochondrial Reactive Oxygen Species in Spinal Ligament-Derived Cells from Ossification of Posterior Longitudinal Ligament Patient

Jong Tae Kim [1,†], Yonggoo Kim [2,†], Ji Yeon Kim [3], Seungok Lee [3], Myungshin Kim [2] and Dong Wook Jekarl [2,*]

[1] Department of Neurosurgery, Incheon St. Mary's Hospital, College of Medicine, The Catholic University of Korea, Seoul 06591, Republic of Korea
[2] Department of Laboratory Medicine, Seoul St. Mary's Hospital, College of Medicine, The Catholic University of Korea, Seoul 06591, Republic of Korea
[3] Department of Laboratory Medicine, Incheon St. Mary's Hospital, College of Medicine, The Catholic University of Korea, Seoul 06591, Republic of Korea
* Correspondence: bonokarl@catholic.ac.kr; Tel.: +82-(02)-22581643; Fax: +82-(02)-22581649
† These authors contributed equally to this work.

Supplementary Table S1. Summarization of raw reads statistics by RNA-seq with base quality scores.

	Total read bases	Total read pairs	GC conent	% of >=Q30 Bases *	Mean Quality Score *
Control1	8,655,845,238	85,701,438	50.79	89.27	34.90
Control3	8,239,947,438	81,583,638	51.50	88.89	34.78
Control4	8,335,764,724	82,532,324	50.99	89.28	34.89
Control5	10,524,839,734	104,206,334	50.44	92.13	35.50
Control6	10,013,411,892	99,142,692	50.80	92.31	35.54
Average	9,153,961,805	90,633,285	50.79	90.38	35.12
OPLL1	7,683,661,254	76,075,854	50.47	89.32	34.91
OPLL2	8,983,418,336	88,944,736	50.24	89.93	35.09
OPLL3	10,285,670,118	101,838,318	50.31	88.22	34.63
OPLL4	10,301,625,290	101,996,290	50.21	89.85	35.07
OPLL5	9,298,691,250	92,066,250	50.38	89.51	34.97
OPLL6	9,583,903,130	94,890,130	50.38	89.31	34.90
Average	9,356,161,563	92,635,263	50.39	89.36	34.93

Control, cervical herniated nucleus pulposus; OPLL, ossification of posterior longitudinal ligament.
* The Quality socres are based on Phred score

Supplementary Table S2. Statistical summary of assembled and aligned raw read against human genome UCSC hg19 using Tophat2.

Patients	Control 1	Control 3	Control 4	Control 5	Control 6	OPLL1	OPLL2	OPLL3	OPLL4	OPLL5	OPLL6
Reads, Left	41429327	39215069	39715062	50315359	47959586	36578191	42860959	48758292	49207084	44357435	45685400
Mapped Reads,Left	40439945	38099939	38906245	48931075	46632390	35469873	41491780	47285628	47738877	43063966	44268694
(%)	97.6	97.2	97.0	97.2	97.2	97.0	96.8	97.0	97.0	97.1	96.9
Multiple aligned,Left	3407494	3246884	3239892	4244524	3945722	2961213	3312407	3717896	4040318	3630437	3670213
(%)	8.5	8.5	8.4	8.7	8.5	8.3	8	7.9	8.5	8.4	8.3
Reads, Right	41248258	39215069	39715062	50315359	47959586	36578191	42860959	48758292	49207084	44357435	45685400
Mapped Reads,Right	39986082	38075237	38500961	48866005	46579867	35435179	41482002	47258716	47724755	43030761	44214093
(%)	96.9	97.1	96.9	97.2	97.1	96.9	96.8	96.9	97.0	97.0	96.8
Multiple aligned,Right	3406135	3247294	3240184	4243273	3944557	2960615	3313339	3718349	4041287	3628994	3668169
(%)	8.5	8.5	8.4	8.7	8.5	8.4	8.0	7.9	8.5	8.4	8.3
Overall mapping Rate	97.0	97.1	97.0	97.2	97.2	96.9	96.8	97.0	97.0	97.0	96.8
Aligned pairs	39101729	37212751	37604622	47908348	45651639	34642694	40512336	46170456	46636014	42098891	43181832
Multiple Aligned pairs	3324940	3169429	3161399	4147794	3858287	2889411	3227568	3718349	3941778	3545133	3574156
Discordant Aligned pairs	1188673	1037233	1196774	1548755	1422772	1146259	1223671	1439973	1536170	1501052	1414081
Concordant Alignment (%)	91.9	92.2	91.7	92.1	92.2	91.6	91.7	91.7	91.7	91.5	91.4

Control, cervical herniated nucleus pulposus; OPLL, ossification of posterior longitudinal ligament.

Supplementary Table S3. The number of very low, low, high and very high transcripts in FPKM values.

sample	FPKM Bins <1	FPKM Bins 1-10	FPKM Bins 11-100	FPKM Bins >100
Control1	35507	8941	3945	495
Control3	35795	8625	3995	513
Control4	35742	8657	3942	547
Control5	35554	8591	4172	571
Control6	35850	8511	4004	523
OPLL1	35482	8651	4192	563
OPLL2	35654	8553	4121	560
OPLL3	35761	8484	4091	552
OPLL4	35702	8472	4141	573
OPLL5	35736	8290	4267	595
OPLL6	35605	8454	4240	589

Control, cervical herniated nucleus pulposus; OPLL, ossification of posterior longitudinal ligament; FPKM, fragments per kilobase of exon per million fragments mapped.

Supplementary Table S4. Genes with highest transcript abundance by FPKM values in Control group.

	FPKM Bins Control1		FPKM Bins Control3		FPKM Bins Control4		FPKM Bins Control5		FPKM Bins Control6
ACTB	3979	*COL1A2*	3532	*ACTB*	4359	*ACTB*	3542	*ACTB*	4578
COL1A2	3515	*LGALS1*	2904	*IGFBP3*	4347	*GAPDH*	3445	*TMSB4X*	3695
GAPDH	3085	*GAPDH*	2582	*TMSB4X*	4157	*SNORD50A*	3244	*ACTG1*	3441
ACTG1	2805	*FTH1*	2525	*LGALS1*	3152	*ACTG1*	3228	*LGALS1*	3367
RPL41	2538	*RPL41*	2402	*ACTG1*	3151	*LGALS1*	3167	*GAPDH*	2965
LGALS1	2510	*FTL*	2387	*COL1A2*	3075	*TMSB4X*	3119	*COL1A2*	2896
TMSB4X	2371	*ACTB*	2377	*GAPDH*	2680	*RPL41*	3081	*TMSB10*	2881
FTL	2245	*TGFBI*	2055	*RPL41*	2587	*TMSB10*	2998	*RPL41*	2683
TMSB10	2244	*VIM*	2011	*TMSB10*	2455	*IGFBP3*	2491	*VIM*	1938
S100A6	1948	*S100A6*	1921	*RPLP1*	2252	*RPLP1*	2066	*RPLP1*	1917
RPLP1	1898	*RPLP1*	1919	*RPS2*	1919	*VIM*	2035	*RPS2*	1788
FTH1	1895	*SERPINE2*	1880	*EEF1A1*	1828	*COL1A2*	1844	*ANXA2*	1781
VIM	1872	*ACTG1*	1849	*S100A6*	1703	*S100A6*	1747	*TGFBI*	1624
IGFBP3	1862	*TIMP1*	1839	*RPS12*	1531	*RPS2*	1745	*RPS27*	1550
ANXA2	1755	*EEF1A1*	1808	*VIM*	1520	*RPS12*	1644	*RPS12*	1451

Supplementary Table S5. Genes with highest transcript abundance by FPKM values in OPLL group.

	FPKM Bins OPLL1		FPKM Bins OPLL2		FPKM Bins OPLL3		FPKM Bins OPLL4		FPKM Bins OPLL5		FPKM Bins OPLL6
ACTB	4888	*ACTB*	5012	*ACTB*	5366	*ACTB*	5573	*ACTB*	7305	*ACTB*	5044
LGALS1	3801	*ACTG1*	3642	*ACTG1*	3572	*TMSB10*	3957	*SNORD65*	5457	*COL1A1*	3672
TMSB4X	3722	*TMSB10*	3383	*GAPDH*	3470	*ACTG1*	3627	*ACTG1*	4376	*GAPDH*	3563
ACTG1	3692	*COL1A1*	3362	*COL1A1*	3368	*LGALS1*	3607	*GAPDH*	4370	*ACTG1*	3500
GAPDH	3382	*GAPDH*	3236	*LGALS1*	3157	*GAPDH*	3551	*LGALS1*	3220	*TMSB10*	3484
RPL41	3161	*TGFBI*	3218	*TMSB4X*	3124	*COL1A1*	3533	*VIM*	3105	*LGALS1*	3453
TMSB10	3059	*ACTB*	3173	*RPL41*	2707	*TMSB4X*	3445	*TMSB10*	3079	*RPL41*	2948
COL1A1	3022	*TMSB4X*	3029	*TAGLN*	2280	*RPL41*	3090	*RPL41*	3071	*TMSB4X*	2929
VIM	2153	*RPL41*	2803	*TGFBI*	2221	*TAGLN*	2823	*TAGLN*	2908	*TGFBI*	2120
COL1A2	2101	*EEF1A1*	2492	*TMSB10*	1703	*TGFBI*	2668	*TMSB4X*	2520	*RPS2*	2024
TGFBI	1986	*TAGLN*	1992	*COL1A2*	1615	*RPS2*	1855	*RPS2*	1960	*COL1A2*	1986
ANXA2	1942	*COL1A2*	1770	*RPLP1*	1612	*RPLP1*	1814	*RPLP1*	1916	*MT2A*	1953
RPS2	1883	*ANXA2*	1737	*ANXA2*	1600	*RPL37A*	1636	*THBS1*	1775	*RPLP1*	1931
MT2A	1837	*RPS2*	1635	*RPS2*	1541	*COL1A2*	1632	*CFL1*	1692	*TAGLN*	1836
S100A6	1832	*RPLP1*	1579	*RPL37A*	1503	*RPLP0*	1503	*MYL6*	1625	*ANXA2*	1671

Supplementary Table S6. Spearman's Correlation matrix of transcript expression between Control and OPLL patients.

	Control1	Control3	Control4	Control5	Control6	OPLL1	OPLL2	OPLL3	OPLL4	OPLL5	OPLL6
Control1	1	0.81093878	0.82392716	0.81568936	0.81346282	0.80797621	0.80754093	0.8006965	0.7990753	0.79437231	0.80519325
Control3	0.81093878	1	0.81049698	0.801077	0.7994192	0.79886441	0.79858701	0.78604085	0.79177608	0.7772218	0.79743873
Control4	0.82392716	0.81049698	1	0.81953503	0.82271392	0.81130226	0.8135719	0.80388758	0.80543476	0.79517516	0.80967164
Control5	0.81568936	0.801077	0.81953503	1	0.82152901	0.81271211	0.81641634	0.80734494	0.81059557	0.79882674	0.81552424
Control6	0.81346282	0.7994192	0.82271392	0.82152901	1	0.81112485	0.81605847	0.80621784	0.80872815	0.79987444	0.81331484
OPLL1	0.80797621	0.79886441	0.81130226	0.81271211	0.81112485	1	0.82023349	0.81041636	0.80910271	0.80603694	0.81822851
OPLL2	0.80754093	0.79858701	0.8135719	0.81641634	0.81605847	0.82023349	1	0.81739267	0.82207437	0.81002571	0.82372707
OPLL3	0.8006965	0.78604085	0.80388758	0.80734494	0.80621784	0.81041636	0.81739267	1	0.81619828	0.80853804	0.81668582
OPLL4	0.7990753	0.79177608	0.80543476	0.81059557	0.80872815	0.80910271	0.82207437	0.81619828	1	0.8056193	0.81790735
OPLL5	0.79437231	0.7772218	0.79517516	0.79882674	0.79987444	0.80603694	0.81002571	0.80853804	0.8056193	1	0.80993505
OPLL6	0.80519325	0.79743873	0.80967164	0.81552424	0.81331484	0.81822851	0.82372707	0.81668582	0.81790735	0.80993505	1

Supplementary Table S7. Differential expression gene by FDR from number 1 to 210.

1	ICAM1	PAPPA2	RANBP17	THSD7B	KIAA1644	PGM1	SH3BP1
2	TMEFF2	CFI	SH3TC1	SLC22A15	NID2	PRSS12	EBP
3	DDIT4	DCN	SEMA3C	GRIN2D	GLYATL2	CDCA2	NINJ1
4	MOCOS	RASSF9	PLAC9	SLC2A12	SLFN5	CCNE2	TXNIP
5	APOL6	FOXC1	PRKAA2	NTNG2	HMGB1	DKK3	LOC100049716
6	SCRG1	FLJ35024	MAP2	DHRS3	CHAC1	TYMS	CHAF1A
7	TMSB15A	COL15A1	SLC1A5	DNAJC9	KIAA1524	SHCBP1	H2AFZ
8	NES	STC1	HEG1	CLEC11A	GJD3	MAD2L1	GPR124
9	FNDC1	METTL7B	AKNA	SLC6A17	COL12A1	BRIP1	UBASH3B
10	CARD11	CFB	PSAT1	LBX2-AS1	ADAM19	MANF	CENPH
11	SIPA1L2	NEFM	STK17B	B4GALNT1	ITGA11	TRIB3	C11orf63
12	SDK2	ZNF521	LRRN4CL	ANKRD33B	DOCK5	CHTF18	FTH1
13	C1R	LINC00968	F2R	NFE2L3	PLEKHG2	LOC728554	GINS2
14	FOXL1	ODF3B	INE2	GPC6	LGALS3BP	TMSB15B	POLA2
15	KCNJ8	FMNL1	COL22A1	RPS10	TIAM2	ABHD4	C15orf59
16	KIF26B	OPLAH	PAX9	SLC1A1	A1BG	ERCC6L	SPHK1
17	FAM129A	FOXF1	MICALCL	SLC16A12	PHLDA2	METRNL	RIPK4
18	PPL	MLLT11	ZYG11A	FSD1	CREBRF	RP11-224O19.2	GPT2
19	ANGPTL5	CELSR1	CLIC3	FZD7	LOC100131315	BDKRB2	JMY
20	ANK3	PRDM6	SLC7A5	FLJ46906	MKNK2	NLGN1	KIF14
21	SESN2	CA5B	SFRP4	ACSS3	NFKBIA	ULBP1	TAPBPL
22	CYP27A1	CEBPD	PTGS2	SCN1B	CCDC34	CMC2	MTRNR2L9
23	TSPAN11	DCHS1	EPS8L2	CTSD	GLIPR1	GBP1	TIMELESS
24	CNN1	PLEKHF1	EFNB2	A1BG-AS1	RRM1	CHRNA5	FOXC2-AS1
25	ACKR3	SPINT2	SOD2	TRIM47	TSC22D3	CDA	MYO1D
26	CPXM2	IGFBP4	MAB21L2	DRAM1	ITGBL1	PPP1R14C	TRIP13
27	FOXO1	WISP1	PTPRG	CPQ	SMS	RPL22L1	TONSL
28	SLC26A10	RASIP1	CEBPB.AS1	ZNF702P	ZNF469	HSPE1	CCNF
29	ARC	ABCC3	CD55	CBX2	FEZ1	KIF11	DENND6B
30	FOXF2	CYTIP	GALNT3	FEN1	CCDC122	FOXS1	ATAD5

Supplementary Table S8. Differential expression gene by FDR from number 211 to 392.

211	UBE2S	THY1	RFC3	CHAC2	LINC00842	RPS29	LOXL3
212	ZNF93	MTRNR2L2	VRK1	PSTPIP1	FAT1	ERV3-1	DNASE2
213	LIN7A	NYAP1	CCDC152	TMEM47	FOXC2	CCDC150	
214	SPAG5	C1orf110	MTRNR2L8	FAM110B	DEPDC1	COPZ2	
215	TBC1D8	TUBA1B	TIMM10	TMEM30B	KIAA1462	PLXNA1	
216	NCAPD3	AGMO	HHIPL1	DCLRE1B	COL5A2	LDLRAD2	
217	RTTN	LOC101927051	DONSON	SLC38A2	CYP4V2	PSD3	
218	LOC283070	AKAP12	MTURN	FANCD2	ESCO2	INADL	
219	S1PR3	MZT1	BDKRB1	ZDHHC1	FANCM	ZNF33B	
220	FRRS1	FAM167B	C11orf82	KIAA1614	HDAC4	GLS	
221	POLE	APOBEC3B.AS1	GPR153	FANCG	PLBD1	STXBP5.AS1	
222	HS3ST3B1	ADIRF	TSNARE1	BUB1B	EIF5A	RAP2B	
223	DNER	OTUD3	PDE1A	ATP9A	LRP1	PIGZ	
224	PLEK2	B4GALT1	C5orf34	TPX2	SAMD9L	SH3KBP1	
225	SLC3A2	FABP5	IRS2	C9orf3	MALAT1	C16orf86	
226	RARRES3	TMEM154	PELI2	COL1A2	FGF2	CDC42EP5	
227	RECQL4	CKS2	ABCC1	GREM1	SLCO3A1	FERMT1	
228	NBR2	C7orf31	LHFP	CXCL12	KIF15	ZC3H6	
229	GINS4	UBE2T	LTBP1	NNMT	CD81	CAMK1D	
230	KIFC1	KRT34	GLTSCR2	PDE5A	FTL	ARL4D	
231	GDF15	TUBE1	NANOS1	AGAP11	HEXA	PTPLAD2	
232	GDF5	DAPK2	RPA3	SGOL1.AS1	MYO7B	RNF208	
233	XPOT	NAALADL2	LBX2	TPP1	RAB3A	SLC31A2	
234	RBP4	CDCA5	RP11-80G7.1	FUCA1	SELM	WHAMMP2	
235	GLA	MTMR9LP	PLCD3	FABP3	BST1	RPSAP52	
236	CENPI	TMPO	C4orf21	NDC80	HCG4B	SNAPC1	
237	TCP11L2	TK1	COL3A1	DBF4	CCDC15	DNAH1	
238	HAUS5	CBR3	DPP7	CEP128	FRAT1	RAB11FIP5	
239	C1orf112	SPDL1	HSBP1L1	PKNOX2	ARHGEF5	FAM210B	
240	ZNF385D	E2F1	SPC25	PRR11	IL8	VAT1	

Supplementary Table S9. Gene set enrichment analysis for 392 differential expression gene**.**

Gene Set Enrichment Analysis	Number of gene	FDR<0.05
CELL CYCLE PHASE	11	0.003119197
MITOTIC CELL CYCLE	12	0.007569438
INTRACELLULAR NONMEMBRANE BOUND ORGANELLE	17	0.007758451
NONMEMBRANE BOUND ORGANELLE	17	0.006651832
CELL CYCLE	19	0.006095019
CELL CYCLE PROCESS	12	0.009286976
INTRACELLULAR ORGANELLE PART	24	0.007468865
ORGANELLE PART	24	0.005955599
CHROMOSOME	10	0.016015256
DNA METABOLIC PROCESS	10	0.046690717

Supplementary Table S10. DAVID Gene set enrichment analysis for 392 differential expression gene**.**

Functional Annotation Cluster	No. of gene	FDR<0.05	Genes
DNA metabolic process	27	0.018046848	*HMGB1, DBF4, POLA2, RPA3, TK1, CCNE2, TYMS, FANCM, FANCG, FEN1, TRIP13, RECQL4, GINS2, GINS4, POLE, BRIP1, ESCO2, SOD2, JMY, DNASE2, RFC3, DCLRE1B, FANCD2, RRM1, CHTF18, CHAF1A, IGFBP4*
DNA replication	15	0.049312643	*HMGB1, GINS2, DBF4, POLE, GINS4, POLA2, TK1, RPA3, CCNE2, TYMS, RFC3, RRM1, CHTF18, CHAF1A, FEN1*
DNA replication	10	0.041944312	*GINS2, RFC3, DBF4, RRM1, POLE, GINS4, CHTF18, POLA2, CHAF1A, RPA3*
Cell cycle	26	0.001630114	*E2F1, KIFC1, HAUS5, DBF4, CCNE2, SPC25, CDCA2, C11ORF82, CDCA5, ERCC6L, TXNIP, KIF11, CCNF, NDC80, ESCO2, NCAPD3, FSD1, MAD2L1, TIMELESS, FANCD2, SPAG5, CKS2, CHTF18, BUB1B, CHAF1A, UBE2S*
Mitosis	15	0.008805729	*KIFC1, HAUS5, KIF11, CCNF, NDC80, NCAPD3, FSD1, SPC25, MAD2L1, TIMELESS, SPAG5, CDCA2, BUB1B, CDCA5, ERCC6L*
Cell division	18	0.009460625	*KIFC1, HAUS5, KIF11, CCNF, NDC80, NCAPD3, FSD1, CCNE2, SPC25, MAD2L1, TIMELESS, SPAG5, CDCA2, CKS2,BUB1B, CDCA5, UBE2S, ERCC6L*
Nuclear division	17	0.015600154	*KIFC1, HAUS5, KIF11, CCNF, KIF15, TPX2, NDC80, NCAPD3, FSD1, SPC25, MAD2L1, TIMELESS, SPAG5, CDCA2, BUB1B, CDCA5, ERCC6L*
Mitosis	17	0.015600154	*KIFC1, HAUS5, KIF11, CCNF, KIF15, TPX2, NDC80, NCAPD3, FSD1, SPC25, MAD2L1, TIMELESS, SPAG5, CDCA2, BUB1B, CDCA5, ERCC6L*
M phase of mitotic cell cycle	17	0.019628135	*KIFC1, HAUS5, KIF11, CCNF, KIF15, TPX2, NDC80, NCAPD3, FSD1, SPC25, MAD2L1, TIMELESS, SPAG5, CDCA2, BUB1B, CDCA5, ERCC6L*
Organelle fission	17	0.025895952	*KIFC1, HAUS5, KIF11, CCNF, KIF15, TPX2, NDC80, NCAPD3, FSD1, SPC25, MAD2L1, TIMELESS, SPAG5, CDCA2, BUB1B, CDCA5, ERCC6L*
Cell cycle process	28	0.044324081	*E2F1, KIFC1, HAUS5, DBF4, SESN2, SPC25, CDCA2, TUBE1, C11ORF82, CDCA5, TRIP13, ERCC6L, KIF11, IL8, KIF15, CCNF, POLE, TPX2, NDC80, NCAPD3, JMY, FSD1, MAD2L1, TIMELESS, FANCD2, SPAG5, CKS2, BUB1B*
Cell cycle phase	23	0.053017648	*E2F1, KIFC1, HAUS5, KIF11, DBF4, CCNF, KIF15, POLE, TPX2, NDC80, NCAPD3, FSD1, SPC25, MAD2L1, TIMELESS,SPAG5, FANCD2, CDCA2, CKS2, BUB1B, CDCA5, TRIP13, ERCC6L*
Extracellular structure organization	14	0.041505388	*B4GALT1, COL3A1, NLGN1, DCN, COL5A2, ANK3, DNER, FOXF1, FOXF2, COL1A2, COL12A1, FOXC2, FOXC1, F2R*

Supplementary Table S11. GAGE analysis with up regulated gene sets for all the genes**.**

Increased in OPLL	Up regulated gene set in OPLL	FDR	No. gene
hsa03040	Spliceosome	3.64E-20	88
hsa03008	Ribosome biogenesis in eukaryotes	4.76E-13	58
hsa03030	DNA replication	1.76E-09	21
hsa04110	Cell cycle	1.66E-06	85
hsa03050	Proteasome	2.41E-06	32
hsa03013	RNA transport	4.42E-06	98
hsa04114	Oocyte meiosis	3.35E-05	67
hsa03410	Base excision repair	4.61E-05	22
hsa00190	Oxidative phosphorylation	5.44E-05	74
hsa03022	Basal transcription factors	7.76E-04	22
hsa00900	Terpenoid backbone biosynthesis	2.18E-03	11
hsa03440	Homologous recombination	6.25E-03	14
hsa00630	Glyoxylate and dicarboxylate metabolism	6.25E-03	12
hsa03018	RNA degradation	9.00E-03	44
hsa03420	Nucleotide excision repair	9.46E-03	28
hsa00240	Pyrimidine metabolism	1.41E-02	59
hsa00020	Citrate cycle (TCA cycle)	2.41E-02	18
hsa03020	RNA polymerase	8.64E-02	23
hsa03015	mRNA surveillance pathway	8.64E-02	51

OPLL: ossification of posterior longitudinal ligament; FDR, false discovery rate; No, number

Supplementary Table S12. GAGE analysis with down regulated gene sets for all the genes.

Decreased in OPLL	Down regulated gene set in OPLL	FDR	No. gene
hsa00531	Glycosaminoglycan degradation	0.004625519	10
hsa04142	Lysosome	0.007077652	68
hsa00512	Mucin type O-Glycan biosynthesis	0.020279966	17
hsa04350	TGF-beta signaling pathway	0.027259328	58
hsa04972	Pancreatic secretion	0.028722685	43
hsa04974	Protein digestion and absorption	0.028722685	39
hsa00760	Nicotinate and nicotinamide metabolism	0.028722685	13
hsa04380	Osteoclast differentiation	0.028722685	56
hsa00910	Nitrogen metabolism	0.028722685	10
hsa04610	Complement and coagulation cascades	0.032654887	34
hsa04620	Toll-like receptor signaling pathway	0.040576	46

OPLL: ossification of posterior longitudinal ligament; FDR, false discovery rate; No, number

Supplementary Table S13. Electronic medical records from Ctrl and OPLL group

	Disease contorl (N)	OPLL (N)	Disease control (n=8205)	OPLL (n=509)	P value
Age (yr)	8205	487	54.7 ± 13.4	59.5 ± 10.7	<0.001
Female / Male (n)	8205	509	4635 / 3570	159 / 350	<0.001
Height (cm)	7026	461	162.7 ± 8.9	164.9 ± 9.1	<0.001
Weight (kg)	7026	461	63.6 ± 12.2	69.4 ± 13.9	<0.001
BMI (kg/m^2)	7026	461	23.9 ± 3.5	25.4 ± 4.3	<0.001
Surgery (yes, n)	8441	273	129	144	<0.001
White blood cell (10^9/L)	7972	501	6.8 ± 2.0	7.9 ± 2.4	<0.001
Red blood cell (10^{12}/L)	7829	498	4.3 ± 0.4	4.2 ± 0.5	<0.001
Hemoglobin (g/dL)	7970	501	13.8 ± 1.5	13.2 ± 1.6	<0.001
Hematocrit (Hct)	7970	501	39.7 ± 4.2	39.0 ± 4.4	0.002
MCV (fL)	7823	489	91.3 ± 4.1	91.4 ± 4.0	0.035
MCH (pg)	7823	489	30.7 ± 1.6	30.9 ± 1.5	0.035
MCHC %	7823	489	33.6 ± 0.9	33.8 ± 0.8	<0.001
Platelet (10^9/L)	7971	501	233 ± 57	222 ± 57	<0.001
Iron (mcg/dL)	981	61	83.5 ± 39.1	88.4 ± 44.6	NS
Ferritin (ng/mL)	908	56	360 ± 957	368 ± 551	NS
ALP (U/L)	7233	465	58.3 ± 25.5	62.1 ± 27.8	0.004
GGT (U/L)	4866	329	40.2 ± 57.5	50.6 ± 60.7	0.003
AST (U/L)	7925	504	26.2 ± 21.5	28.6 ± 19.8	0.007
ALT (U/L)	7926	504	26.9 ± 33.1	30.3 ± 27.0	0.007
Total bilirubin (mg/dL)	6335	455	0.71 ± 0.29	0.72 ± 0.30	NS
Sodium (mmol/L)	7705	498	141.3 ± 1.9	140.9 ± 2.1	<0.001
Chloride (mmol/L)	6826	455	104.3 ± 2.2	104.1 ± 2.3	NS
Calcium (mg/dL)	5672	414	9.0 ± 0.4	8.8 ± 0.4	<0.001
Potassium (mmol/L)	7704	498	4.28 ± 0.30	4.25 ± 0.29	0.004
Magnesium (mg/dL)	3237	256	2.1 ± 0.1	2.1 ± 0.1	NS
Phosphorus (mg/dL)	5093	344	3.5 ± 0.4	3.4 ± 0.5	<0.001

Supplementary Table S14. Electronic medical records from surgical Ctrl and OPLL group

	OPLL non-surgery (N)	OPLL surgery (N)	OPLL non-surgery (n)	OPLL surgery (n)	P value
Age (yr)	397	90	59.1 ± 11.0	61.3 ± 9.3	NS
Female / Male (n)	365	144	124 / 241	35 / 109	0.034
Height (cm)	349	112	164.8 ± 9.2	165.2 ± 8.5	NS
Weight (kg)	349	112	69.3 ± 14.5	69.8 ± 12.0	NS
BMI (kg/m^2)	349	112	25.4 ± 4.5	25.4 ± 3.5	NS
White blood cell (10^9/L)	388	112	7.6 ± 2.4	8.8 ± 2.2	<0.001
Red blood cell (10^{12}/L)	377	112	4.3 ± 0.5	4.1 ± 0.4	<0.001
Hemoglobin (g/dL)	388	112	13.3 ± 1.6	12.8 ± 1.3	0.002
Hematocrit (Hct)	388	112	39.3 ± 4.5	37.9 ± 3.6	0.001
MCV (fL)	377	112	91.3 ± 3.9	92.1 ± 4.4	0.044
MCH (pg)	377	112	30.8 ± 1.4	31.1 ± 1.7	NS
MCHC %	377	112	33.8 ± 0.8	33.7 ± 0.8	NS
Platelet (10^9/L)	388	112	219 ± 57	230 ± 56	NS
Iron (mcg/dL)	50	11	93.1 ± 45.6	66.9 ± 33.9	NS
Ferritin (ng/mL)	48	8	377 ± 554	311 ± 570	NS
ALP (U/L)	366	99	58.3 ± 25.5	62.1 ± 27.8	NS
GGT (U/L)	260	69	49.1 ± 61.1	56.3 ± 59.6	0.043
AST (U/L)	392	112	28.8 ± 21.6	27.9 ± 11.6	NS
ALT (U/L)	392	112	30.8 ± 29.2	28.5 ± 16.8	NS
Total bilirubin (mg/dL)	353	102	0.72 ± 0.29	0.73 ± 0.34	NS
Sodium (mmol/L)	386	112	141.1 ± 2.1	140.5 ± 1.8	<0.001
Chloride (mmol/L)	343	112	104.3 ± 2.4	103.1 ± 1.7	0.002
Calcium (mg/dL)	315	99	8.0 ± 0.4	8.7 ± 0.4	<0.001
Potassium (mmol/L)	386	112	4.25 ± 0.30	4.24 ± 0.28	NS
Magnesium (mg/dL)	194	62	2.1 ± 0.1	2.1 ± 0.1	NS
Phosphorus (mg/dL)	270	74	3.4 ± 0.4	3.3 ± 0.6	NS

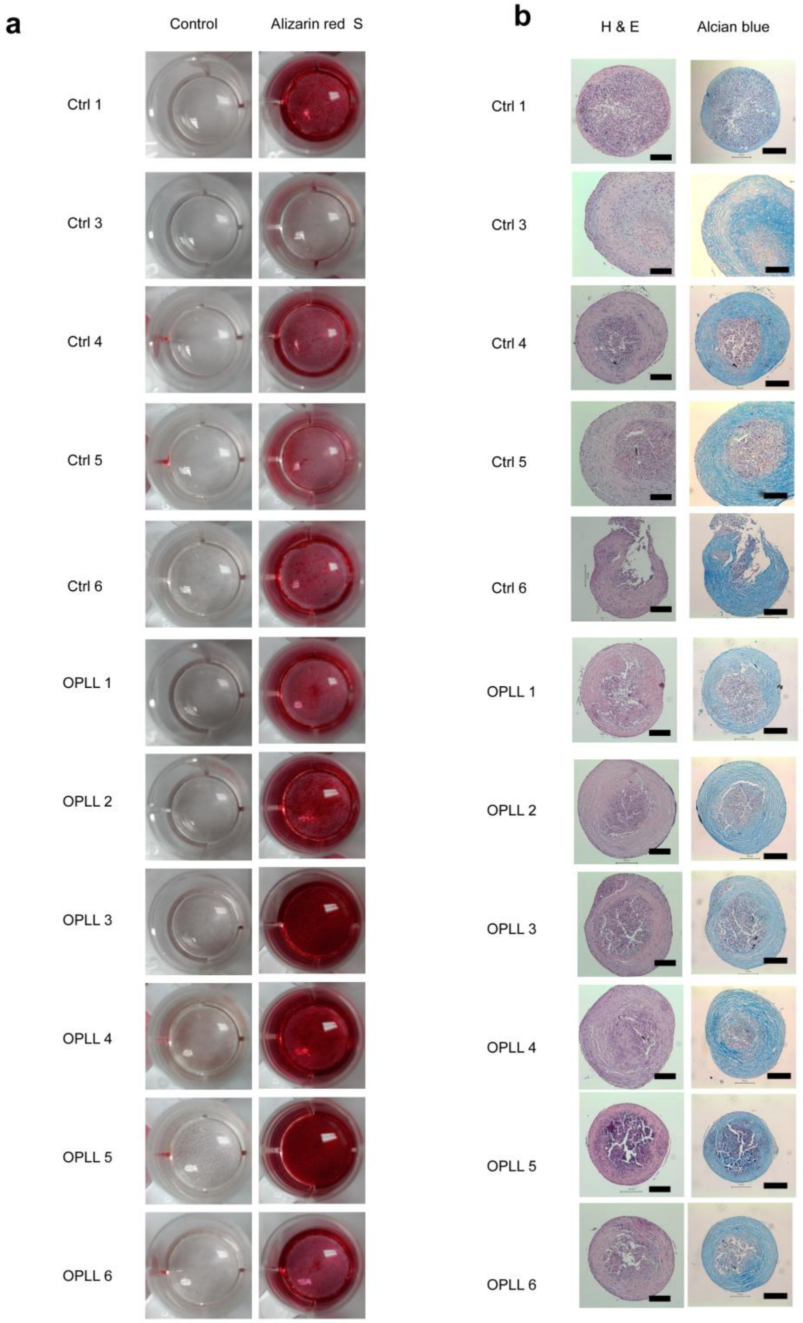

Supplementary Figure S1. Baseline study of SLDCs from control (Ctrl) and OPLL patients. (**a**) Cells from Ctrl and OPLL patients were subjected to osteogenic differentiation for 3 weeks of culture and stained with Alizarin red S (ARS). The data are representative data of triplicate results. (**b**) Chondrogenic differentiation of Ctrl and OPLL patients. The cultured pellet was stained with H&E and Alcian blue reagent. The data are representative data of triplicate results. * P<0.05 within groups. Scale bars: white, 50 μm; black, 100 μm.

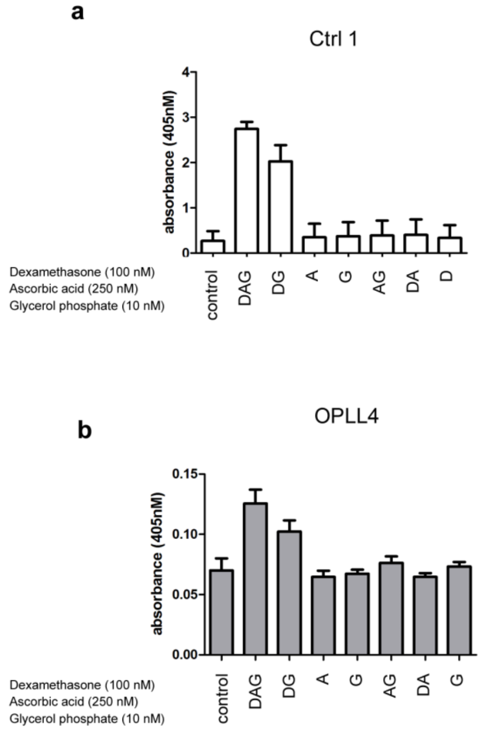

Supplementary Figure S2. Evaluation of osteogenic medium and various molecules. Osteogenic differentiation was tested using a combination of dexamethasone (D), ascorbic acid (A) and β-glycerophosphate (G) for (**a**) Ctrl1 and (**b**) OPLL4. The results showed that the DAG combination was most effective, followed by DG, for osteogenic induction.

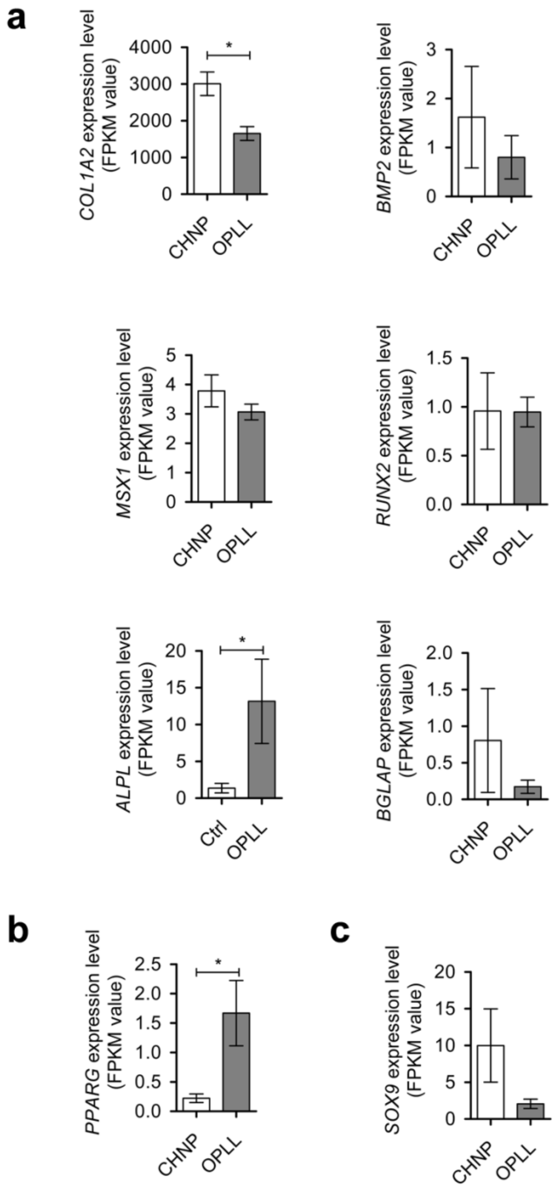

Supplementary Figure S3. mRNA (gene) expression levels from RNA-seq. Baseline gene expression related to (**a**) osteogenesis, (**b**) adipogenesis, and (**c**) chondrogenesis is noted. These gene expression data coincided with the mRNA expression data obtained by qRT–PCR.

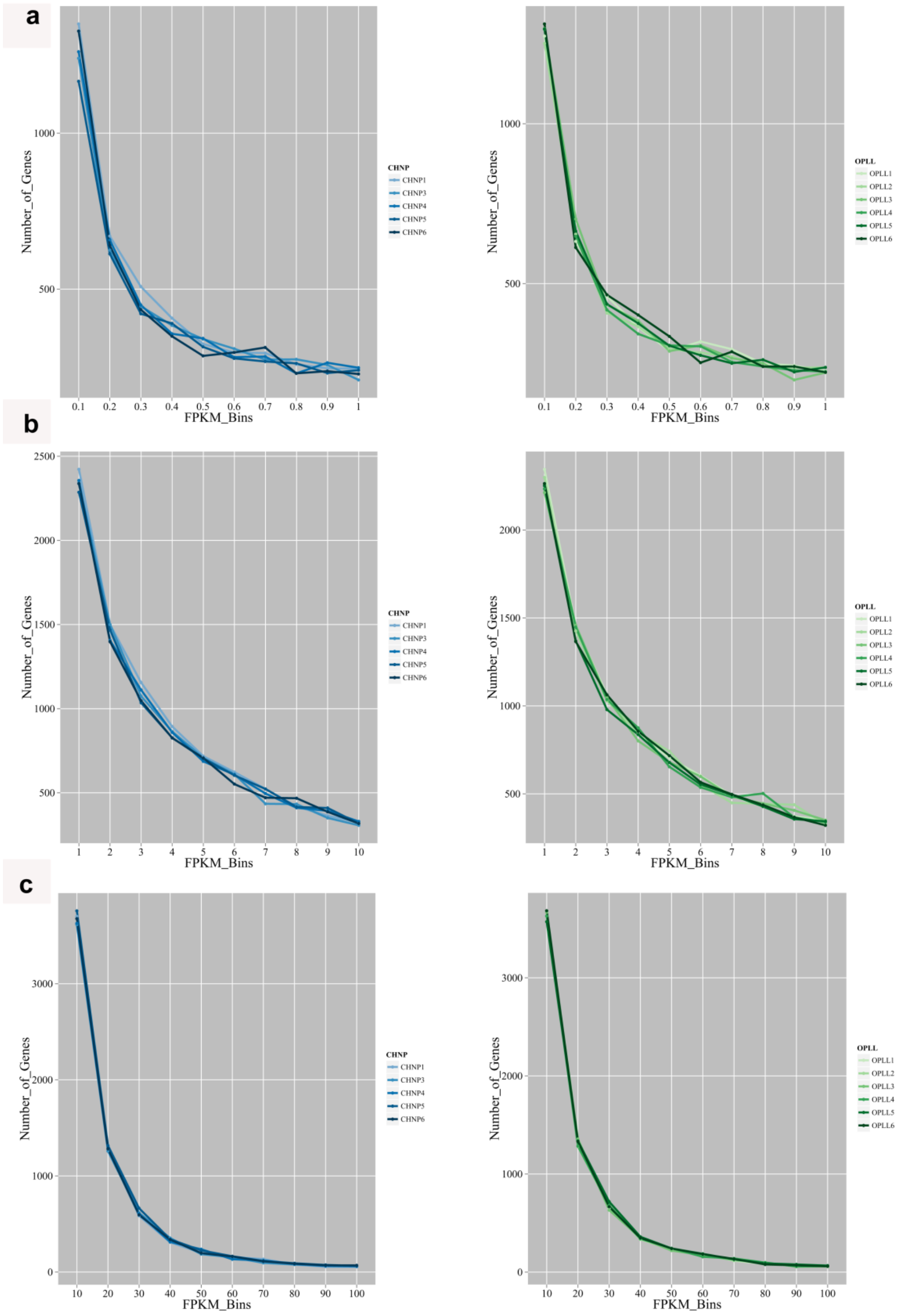

Supplementary Figure S4. Transcript abundance and numbers of genes for control (Ctrl, blue) and OPLL (green) patients. (**a**) Numbers of genes/transcripts with FPKM abundance values below 1. (**b**) Numbers of genes/transcripts with FPKM abundance values between 1 and 10. (**c**) Numbers of genes/transcripts with FPKM abundance values between 10 and 100. FPKM, fragments per kilobase of exon per million mapped fragments.

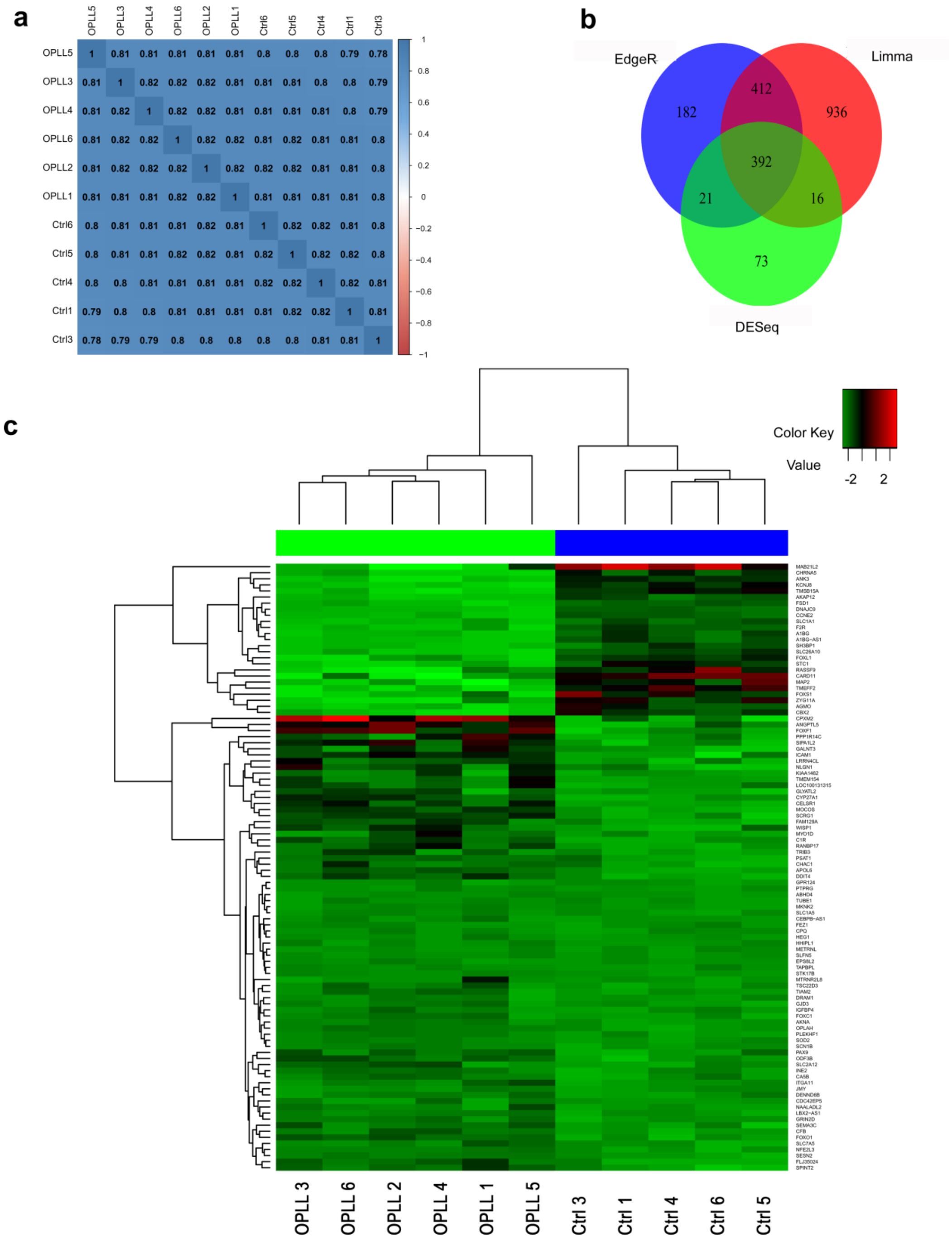

Supplementary Figure S5. Correlations, differentially expressed genes (DEGs) and heatmap from the RNA-seq analysis results. (**a**) Heatmap of the Spearman correlation coefficients matrix between the control (n=5) and OPLL (n=6) groups. The input data were the read counts (FPKM values). (**b**) Differentially expressed genes (DEGs) according to the intersection of EdgeR, Limma and DESeq. The input data were raw read count data, and the analysis resulted in 392 overlapping genes among the DEGs. (**c**) Clustering analysis of 100 representative DEGs among the Ctrl and OPLL groups.

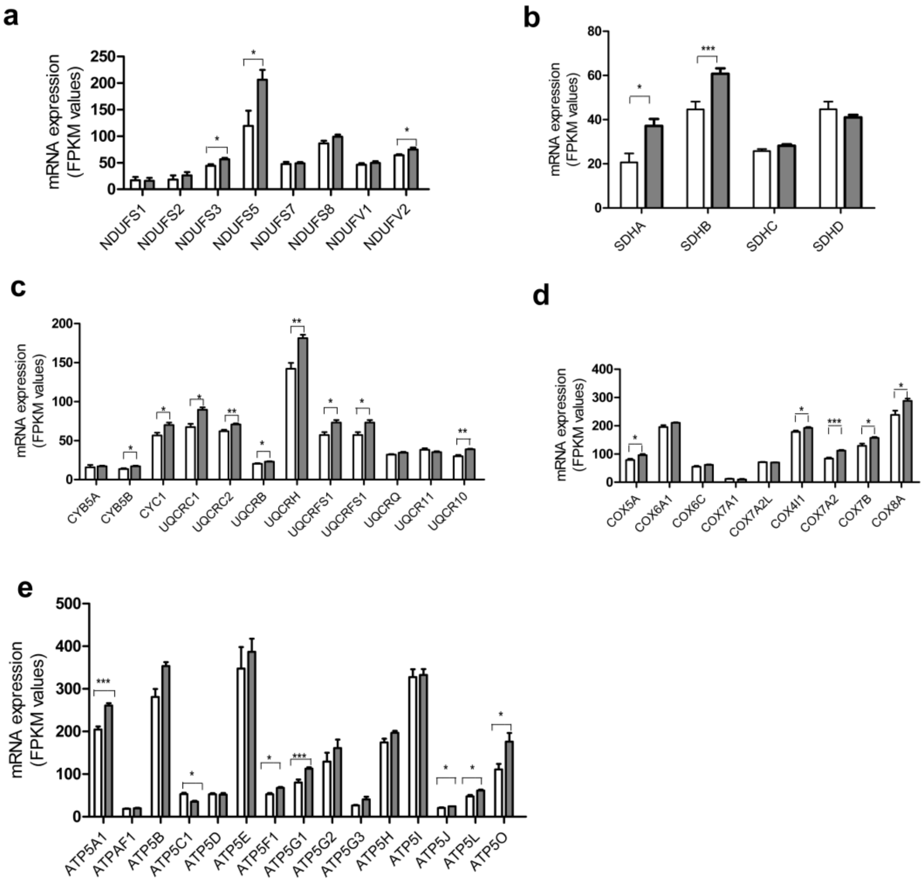

Supplementary Figure S6. mRNA expression of mitochondrial electron transfer chain related complex I ~ V. (a) Complex I related mRNAs. (b) Complex II related mRNAs. (c) Complex III related mRNAs of which CYB5A, CYB5B are mitochondrial origin, whereas other mRNAs are nucleus origin. (d) Complex IV related mRNA. (e) Complex V related mRNA.

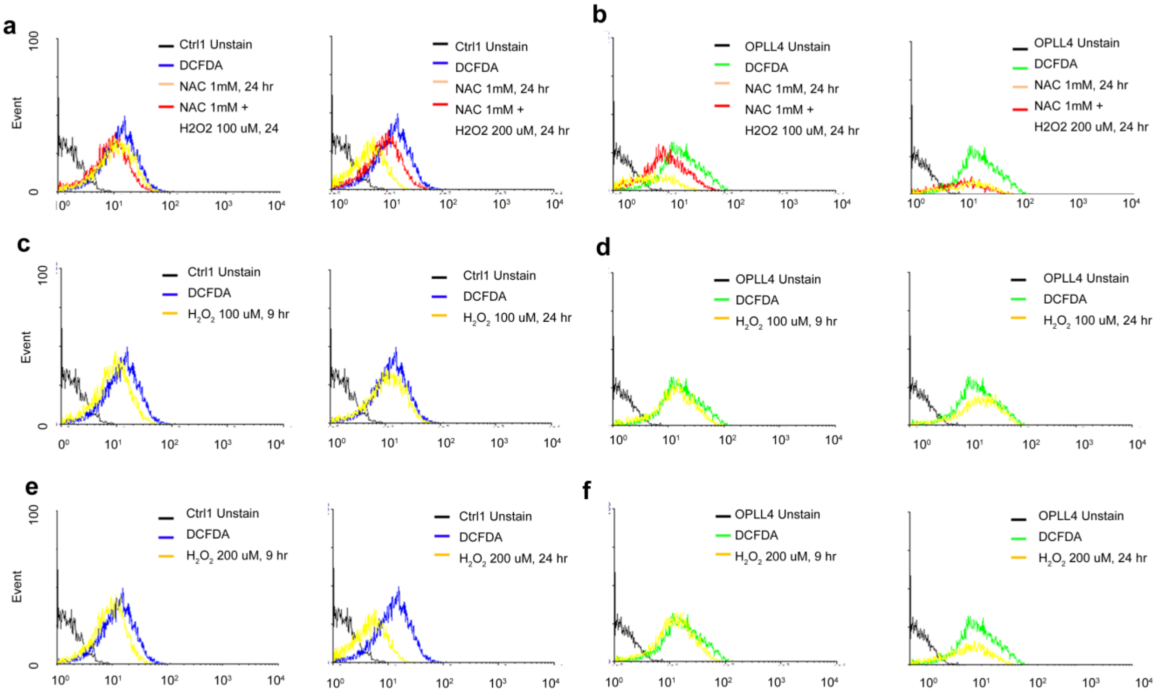

Supplementary Figure S7. Effects of ROS molecules as determined by flow cytometric methods. (**a**) In Ctrl1, H_2O_2 and NAC synergistically repressed ROS as measured by DCFDA over 24 hr. (b) In OPLL4, minimal effects were observed for H_2O_2 and NAC. (c) In Ctrl1, H_2O_2 repressed ROS by 9hr but not by 24 hr. (d) In OPLL4, H_2O_2 repression was not observed. (e) In Ctrl1, 200 nM H_2O_2repressed ROS by 24 hr. (f) In OPLL4, repression was not observed.

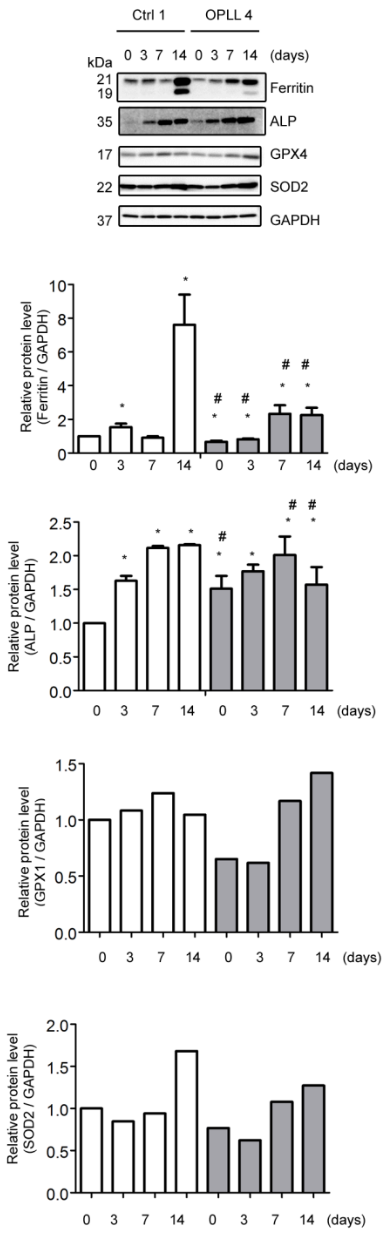

Supplementary Figure S8. Osteogenic differentiation and expression of various proteins. Upon osteogenic differentiation, at Day 14, ferritin, GPX1 and SOD2 levels were increased, which was expected to clear ROS. ALP levels were increased from Day 3. Western blotting of GPX1 and SOD2 was performed only once. * *P*<0.05 within groups; # *P*<0.05 between groups. Scale bars: white, 50 μm; black, 100 μm.

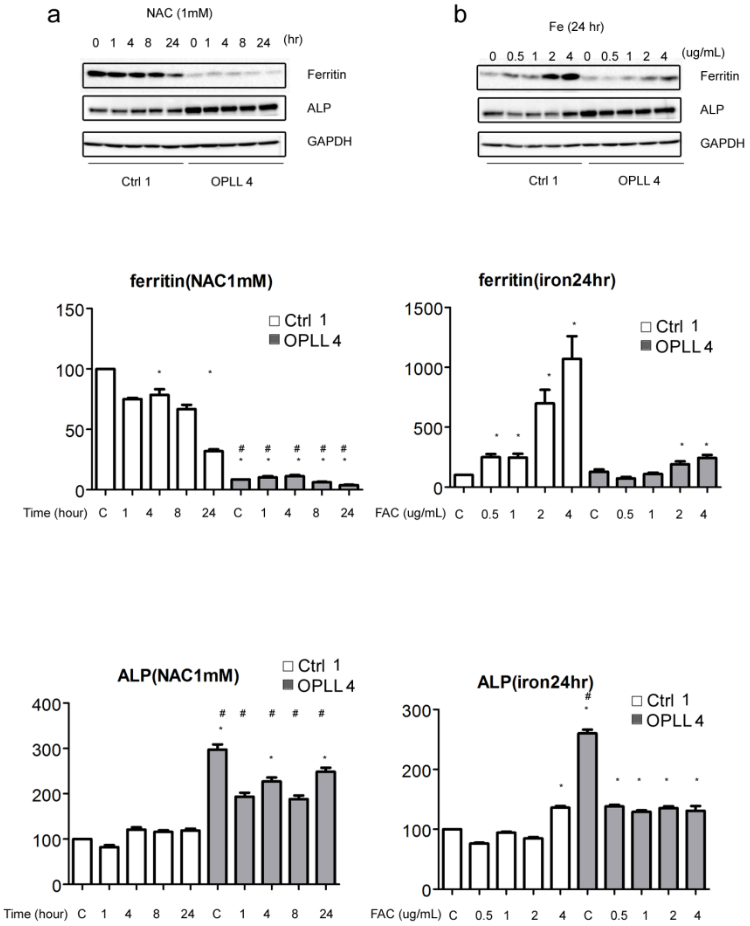

Supplementary Figure S9. Association between ferritin and ROS molecules as determined by Western blotting. (**a**) Addition of 1 mM N-acetyl-cysteine (NAC) abrogated the ferritin level in Ctrl1 and caused a slight decrease in OPLL4, whose baseline ferritin level was low. An increase in the ALP level was not noted in Ctrl1, but the ALP level was slightly decreased in OPLL4. (**b**) Addition of Fe increased ferritin levels in a dose-dependent manner. The ALP level was decreased in OPLL4. * *P*<0.05 within groups; # *P*<0.05 between groups. Scale bar: white, 50 μm; black, 100 μm.

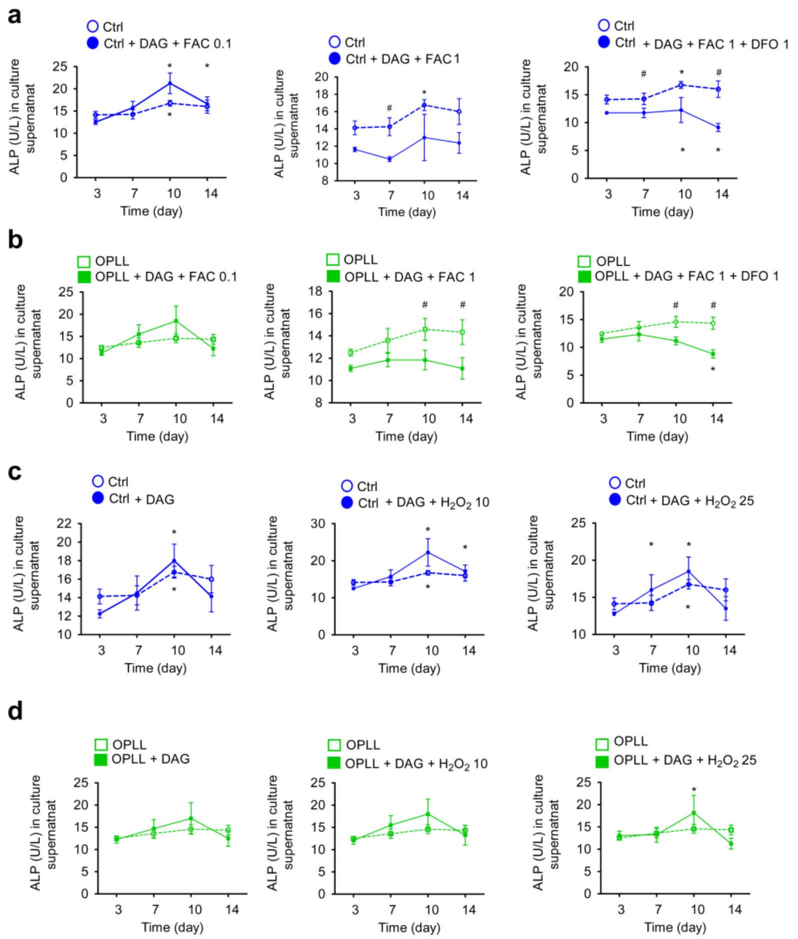

Supplementary Figure S10. Supernatant ALP measurement during osteogenic differentiation. (**a, b**) In the Ctrl and OPLL groups, supernatant ALP levels were increased at Day 10 after addition of 0.1 μg/mL Fe but decreased after addition of 1 μg/mL Fe and 1 μM deferoxamine (DFO). Analysis of ALP in the supernatant of the culture medium demonstrated that the ALP level was lower in the OPLL group than in the Ctrl group from Day 3 to Day 10. A reduced ALP concentration was shown to be present in the supernatant of the OPLL group treated with 1 μg/mL Fe. (**c**) In the Ctrl group, addition of 10 nM H_2O_2 significantly increased the ALP level. (**d**) In the OPLL group, addition of H_2O_2 caused no difference except for H_2O_2 25 nM at 10 days of osteogenic differentiation. * indicates $P<0.05$ within the group; # indicates $P<0.05$ compared to the Ctrl and OPLL groups.

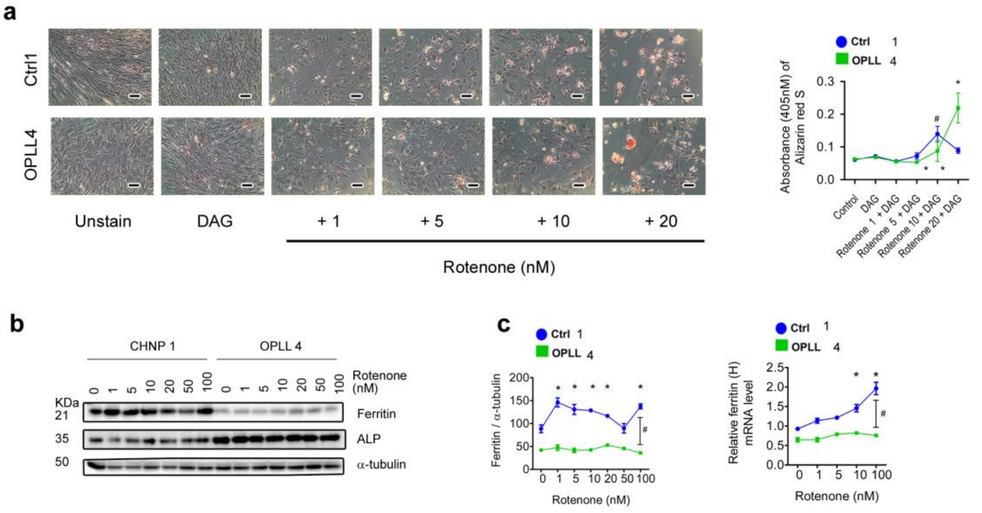

Supplementary Figure S11. Rotenone and osteogenic differentiation.

Rotenone caused slight ROS production by blocking the mitochondrial complex I protein, which is an electron transport chain (ETC) complex protein. (**a**) One, 5, 10, and 20 μM rotenone was added along with DAG for 12 days for osteogenic differentiation. As rotenone is toxic, the incubation was performed until osteogenic differentiation could be investigated. Osteogenesis was induced by rotenone only after the addition of a lethal dose (≥5 nM). (**b**) Ferritin mRNA expression was induced by rotenone addition, but neither ferritin induction nor ALP repression was noted. Rotenone induced ferritin and suppressed ALP at 1 nM, but these effects were not clear at an increased dose. (**c**) Ferritin protein levels increased from 1 nM, but ferritin mRNA levels increased after 1 nM. * P<0.05 within groups; # P<0.05 between groups. Scale bars:white, 50 μm;black, 100 μm.

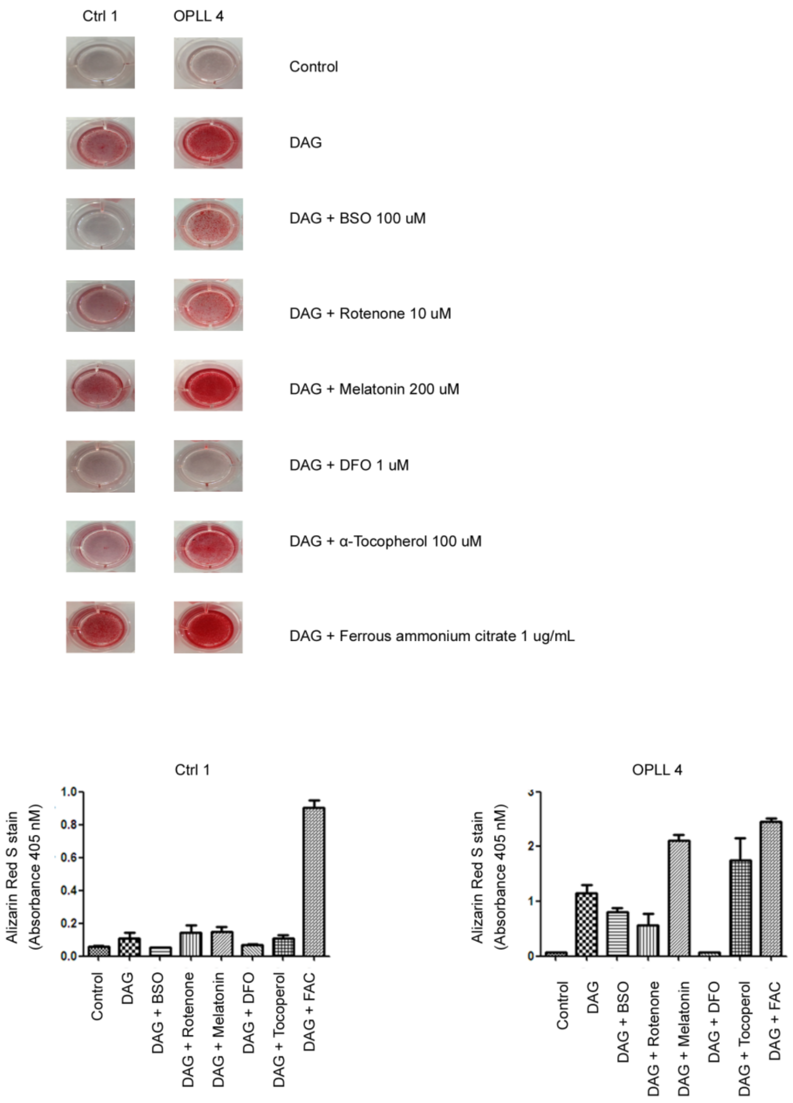

Supplementary Figure S12. Suppression of osteogenic differentiation using various chemicals**.** Addition of BSO (L-buthionine-S, R-sulfoximine; causes ROS overproduction by glutathione synthesis inhibition and GSH depletion), rotenone (causes slight ROS production by mitochondrial complex I blockade), melatonin (scavenges ROS), DFO (deferoxamine; chelates Fe, acts as an antiproliferation agent), tocopherol (scavenges ROS), and Fe (causes ROS production and induces ferritin) for osteogenic suppression revealed that melatonin, α-tocopherol and Fe enhanced osteogenic differentiation. Melatonin andα-tocopherol are known to exhibitanti-ROS functions. ARS staining results from osteogenic differentiation using various chemicals
